# Accurate X-ray Absorption Spectra near L- and M-Edges from Relativistic Four-Component Damped Response Time-Dependent Density Functional Theory

**DOI:** 10.1021/acs.inorgchem.1c02412

**Published:** 2021-12-27

**Authors:** Lukas Konecny, Jan Vicha, Stanislav Komorovsky, Kenneth Ruud, Michal Repisky

**Affiliations:** † Hylleraas Centre for Quantum Molecular Sciences, Department of Chemistry, University of Tromsø − The Arctic University of Norway, 9037 Tromsø, Norway; ‡ Centre of Polymer Systems, Tomas Bata University, tř. Tomáše Bati 5678, 760 01 Zlín, Czech Republic; ¶ Institute of Inorganic Chemistry, Slovak Academy of Sciences, Dúbravská cesta 9, SK-84536 Bratislava, Slovakia

## Abstract

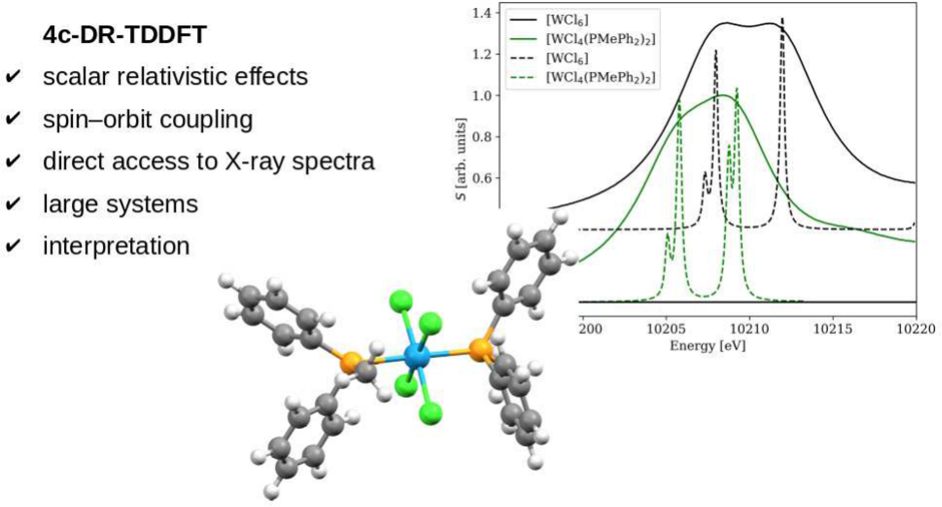

The simulation of X-ray absorption spectra requires both scalar and spin–orbit (SO) relativistic effects to be taken into account, particularly near L- and M-edges where the SO splitting of core p and d orbitals dominates. Four-component Dirac–Coulomb Hamiltonian-based linear damped response time-dependent density functional theory (4c-DR-TDDFT) calculates spectra directly for a selected frequency region while including the relativistic effects variationally, making the method well suited for X-ray applications. In this work, we show that accurate X-ray absorption spectra near L_2,3_- and M_4,5_-edges of closed-shell transition metal and actinide compounds with different central atoms, ligands, and oxidation states can be obtained by means of 4c-DR-TDDFT. While the main absorption lines do not change noticeably with the basis set and geometry, the exchange–correlation functional has a strong influence with hybrid functionals performing the best. The energy shift compared to the experiment is shown to depend linearly on the amount of Hartee–Fock exchange with the optimal value being 60% for spectral regions above 1000 eV, providing relative errors below 0.2% and 2% for edge energies and SO splittings, respectively. Finally, the methodology calibrated in this work is used to reproduce the experimental L_2,3_-edge X-ray absorption spectra of [RuCl_2_(DMSO)_2_(Im)_2_] and [WCl_4_(PMePh_2_)_2_], and resolve the broad bands into separated lines, allowing an interpretation based on ligand field theory and double point groups. These results support 4c-DR-TDDFT as a reliable method for calculating and analyzing X-ray absorption spectra of chemically interesting systems, advance the accuracy of state-of-the art relativistic DFT approaches, and provide a reference for benchmarking more approximate techniques.

## Abstract 2

The paper demonstrates that relativistic four-component TDDFT theory can reproduce and analyze experimental X-ray absorption spectra near L_2,3_- and M_4,5_-edges of transition metal and actinide compounds with different central atoms, ligands, and oxidation states. With variational inclusion of scalar and spin−orbit relativistic effects and hybrid functionals with an optimized amount of Hartee−Fock exchange (60%), it achieves relative errors below 0.2% and 2% for edge energies and spin−orbit (SO) splittings.

## 1 Introduction

X-rays were included in the toolbox of chemists shortly after their discovery by Röntgen^1^ and are used to probe both the molecular structure in diffraction experiments as well as the electronic structure in absorption, emission, and scattering X-ray spectroscopies. The wavelengths of X-rays are comparable to molecular dimensions, allowing the local electronic structure via excitations from localized core orbitals to be probed. X-ray spectroscopies thus offer high spatial resolution as well as elemental sensitivity due to the large energy separation of core levels in different atoms.^2^ In addition, recent years have seen a surge in the development of X-ray instrumentation in the form of fourth-generation synchrotron facilities, including X-ray free-electron lasers producing intense short-duration pulses as well as tabletop X-ray sources via high-harmonic generation.^3,4^ These advances drive the widespread adoption of X-ray spectroscopies and, in turn, prompt the development of computational methods capable of accurately predicting and interpreting both standard and novel X-ray experiments.

In X-ray absorption spectroscopy (XAS), the spectrum is characterized by absorption edges, i.e., abrupt onsets of absorption at resonant energies corresponding to inner-shell excitations. The spectral features close to the absorption edge are part of the near-edge X-ray absorption fine structure (NEXAFS), also known as X-ray absorption near-edge structure (XANES). Higher-energy signals constitute extended X-ray absorption fine structure (EXAFS) spectra that are characterized by weak oscillations originating from resonances in absorptions beyond the ionization energy.^5^ While XANES is used to determine the details of the electronic structure, such as oxidation states, EXAFS provides more information about the geometry around the absorbing center than about the electronic structure. In addition, low-intensity pre-edge peaks arising from excitations to singly occupied molecular orbitals may appear, particularly in transition-metal complexes.^6^ In recent years, techniques for high-resolution XANES^7^ have emerged including high-energy-resolution fluorescence detected XAS (HERFD-XAS),^8,9^ high energy resolution off-resonant spectroscopy (HEROS),^10,11^ and improved detectors.^12−14^

A prerequisite for the reliable quantum-chemical modeling of X-ray spectroscopic processes is the inclusion of relativistic effects defined as differences between the full relativistic description of matter and an approximate Schrödinger equation-based description. This requirement stems from the fact that the inner-shell orbitals involved in XAS processes are most affected by relativity, manifestations of which are frequency shifts of spectral lines due to the scalar relativistic effects as well as spectral fine structure splitting arising from the spin–orbit (SO) coupling.^15−18^ The relativistic effects are significant even in light (third row) elements, highlighting the need for a relativistic description also in these cases,^18^ where perturbative treatment of SO coupling can yield accurate spectra,^19^ and increase in importance for heavier elements.^20^ In K-edge spectra originating from excitations from 1s_1/2_ orbitals, only the scalar relativistic corrections have nonzero contributions and result in a constant shift of K-edge signals. In this case, one-component scalar relativistic Hamiltonians are convenient and sufficient for describing this XAS edge. On the other hand, XAS spectra near L- and M-edges originate from excitations from inner-shell atomic-like p and d orbitals split by the SO interaction into p_1/2_ and p_3/2_ or d_3/2_ and d_5/2_ levels, respectively. As a consequence of the SO splitting, the use of multicomponent, i.e., two-component (2c) or four-component (4c), relativistic Hamiltonians is mandatory to correctly assign X-ray spectra near L- and M-edges.^18,21^ Here, the 4c methodology based on the one-electron Dirac Hamiltonian combined with the instantaneous two-electron Coulomb interaction represents the “gold standard” of relativistic quantum chemistry, since all essential scalar and SO effects are included nonperturbatively.^22^ This is, in particular, important for L-edge XAS, which displays higher resolution due to higher intensities and longer core-hole lifetimes, and is therefore a popular technique in the study of transition metal complexes and solid-state materials.^21^ Further spin-related contributions can be accounted for by the inclusion of the Gaunt term that has been shown to contribute to the energy shifts of core orbitals.^20,23^

When considering electronic structure models, a wide range of methods, including post-Hartree–Fock methods and Kohn–Sham density functional theory (DFT), have been developed and applied to XAS. For a comprehensive account of quantum-chemical methods addressing X-ray spectroscopies, we refer to recent reviews.^24−27^ Since our goal is to target large systems such as heavy-metal complexes, we focus on relativistic DFT that favorably combines computational cost and accuracy. There are two principal categories of DFT for XAS: (a) ΔSCF that adapts ground-state SCF optimization for core-hole electronic configurations;^28^ (b) time-dependent DFT (TDDFT) that describes the response of molecular systems to external X-ray radiation by solving the time-dependent Kohn–Sham (TDKS) equation.^29^ The latter can further be approached in three different ways: real-time TDDFT (RT-TDDFT),^30,31^ linear response TDDFT (LR-TDDFT),^32,33^ and damped response TDDFT (DR-TDDFT).^34−36^

Of these, the RT-TDDFT solves the TDKS equation for a molecule subjected to an external electromagnetic pulse by direct integration in the time domain^37,38^ and has been recently extended into the relativistic domain at both 4c^39,40^ and 2c exact two-component (X2C)^41,42^ level of theory. The spectrum is then obtained by transforming the induced electric dipole moment recorded during the simulation into the frequency domain. While the method holds promise for describing strong-field and time-resolved processes, its relatively high computational cost has so far prevented its widespread use for calculating X-ray spectra even though pioneering studies have been reported at both nonrelativistic^43^ and relativistic levels of theory.^18,44^

The next two approaches are based on perturbation theory. The foundation of LR-TDDFT is an eigenvalue equation (the Casida equation) yielding excitation energies as the eigenvalues and transition amplitudes as the eigenvectors. The linear response to an external field as well as an absorption spectrum can then be evaluated from these excitation energies and transition amplitudes. It is a popular approach due to the availability of efficient algorithms.^45−50^ However, using the well-known Davidson algorithm,^51^ the calculation of excitation energies proceeds from the lowest (valence) excitations, making such an approach to X-ray spectra prohibitively expensive. To mitigate these challenges, core–valence separation (CVS), also referred to as restricted excitation window (REW), techniques were developed in which only a subset of orbitals is considered.^52−59^ It has been shown that CVS causes only negligible errors.^59,60^ Applications of relativistic LR-TDDFT with variational SO interactions and focused on XAS have been reported at the level of the two-component zeroth-order regular approximation (ZORA) Hamiltonian^61^ as well as the X2C Hamiltonian.^62^

Finally, DR-TDDFT (also called the complex polarization propagator (CPP) approach^34−36^) describes the response of molecular systems to electromagnetic radiation directly in the frequency domain while focusing only on the spectral range of interest and, in addition, including relaxation effects by means of a damping parameter. By this means, the full complex response tensor covering both scattering and absorption processes is calculated. As such, DR-TDDFT is readily applicable for high-frequency (X-ray) or high density-of-states spectral regions that may otherwise prove challenging for methods like LR-TDDFT or RT-TDDFT, and so, it is well suited for XAS spectra of large molecules; therefore, it is the method of choice in this work. Here, we rely on our recent implementation of DR-TDDFT at the 4c relativistic level of theory.^63^ Previous XAS applications of the 4c-DR-TDDFT have been reported to study the L_3_-edge of UO_2_^2+^ ^64^ as well as XAS of carbon, silicon, germanium, and sulfur compounds.^65^ In addition, a damped response Bethe–Salpeter equation at the X2C relativistic theory has been recently presented and applied.^66^

When applied to X-ray spectra, DFT is known to suffer from frequency shifts with respect to experimental results, commonly attributed to self-interaction errors.^67^ An established procedure for improving the performance of DFT is to use hybrid exchange–correlation (xc) functionals with an increased amount of exact Hartree–Fock exchange (HFX).^68−70^ Particularly good results have been achieved with BHLYP and B^0.58^LYP, i.e., variants of B3LYP with 50% and 58% of HFX, respectively. In contrast, one study has suggested a smaller amount of HFX.^71^ A similar prescription in range-separated functionals is to increase the asymptotic amount of HFX in CAM-B3LYP from the usual 65% to 100%.^70,72^ Efforts have also been spent on developing special functionals and computational schemes for X-ray spectra, such as short-range corrected functionals,^6,26^ the long- and short-range corrected method LCgau-BOP,^73^ core–valence^74^ and core–valence-Rydberg functionals,^75,76^ or a many-body perturbation theory corrected LR-TDDFT^77^ as well as a combination of restricted open-shell configuration interaction singles (ROCIS) and density functional theory.^78,79^ However, the majority of these studies relied on nonrelativistic, atomic orbital energy-corrected or scalar-relativistic calculations and focused predominantly on K-edges of light elements.

In the present work, we focus on the fully relativistic Dirac–Coulomb Hamiltonian-based 4c-DR-TDDFT level of theory and investigate the role of HFX in global hybrid functionals for reliable relativistic modeling of XAS spectral shifts and shapes. Because our approach is inherently *relativistic* and includes both *scalar and spin–orbit coupling effects variationally*, there is no longer a need to shift the absorption energies to compensate for the lack of relativistic corrections, allowing us to disentangle the contributions from relativity and the electronic structure model and thus avoiding accidental error cancellations. By considering transition metal and actinide compounds with different central atoms, ligands, and oxidation states and calibrating for other calculation parameters, including the molecular geometry and basis set, we propose an optimized computational protocol for calculating XAS spectra near L_2,3_- and M_4,5_-edges using 4c-DR-TDDFT. This is then applied to resolve XAS spectra of larger Ru and W complexes with up to 60 atoms.

The structure of this Article is as follows. Section 2 contains a brief summary of the 4c damped response TDDFT theory. Section 3 presents the computational details, and our results are presented in Section 4, first for the calibration set in Section 4.1 and then for the larger molecular systems in Section 4.2. The paper ends with some concluding remarks in Section 5.

## 2 Theory

The linear response of a molecular system exposed to an external electric field (**E**) of angular frequency (ω) is represented by the complex polarizability tensor (**α**) that parametrizes the induced electric dipole moment [**μ**^ind^(ω)]
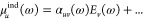
1The tensor **α** is used to evaluate the dipole strength function as (in atomic units)
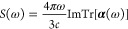
2where *c* is the speed of light, Im denotes the imaginary part of the tensor, and Tr represents the trace over the Cartesian components. The function *S*(ω) defines the electronic absorption spectrum (EAS) across the entire frequency range, including those of relevance to XAS.

In DR-TDDFT, the **α**(ω) tensor components are calculated for a user-defined set of frequencies from the response vectors **X** and **Y** as
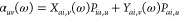
3where **P** is the matrix representation of the electric dipole moment operator. The indices *u* and *v* denote Cartesian components while *a* and *i* run over virtual and occupied reference (ground-state) molecular orbitals (MO), respectively. Note that, in a 4c relativistic theory, index *a* runs in general over virtual positive-energy as well as negative-energy MOs. The response vectors are determined by solving the damped response equation^63,80^
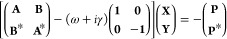
4where ω and γ are user-defined parameters specifying the external electric field frequency and a common relaxation (damping) parameter. The parameter γ models the finite lifetime of the excited states (corresponding to their inverse lifetimes) and leads to finite-width Lorentzian peaks in the spectra (with γ being the half width at half-maximum of the peaks). The right-hand side of eq 4 describes the interaction of the molecular system with the applied external electric field, which in the electric dipole approximation is mediated by the electric dipole moment operator **P**. While the short wavelength of X-ray radiation may require terms beyond the electric dipole,^81−83^ we do not consider this aspect in the present study. Finally, the first term on the left-hand side of eq 4 is the generalized Hessian; for the Dirac–Coulomb Hamiltonian, it is defined by^63^
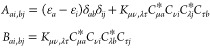
5where ε_*i*_ and ε_*a*_ are the energies of occupied and virtual reference MOs, respectively, δ_*pq*_ is the Kronecker delta function (δ*_pq_* = 1 if *p* = *q* and 0 otherwise), and
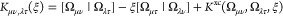
6is the coupling matrix, with *ξ* denoting the admixture of HFX. In the expression for the coupling matrix, [**Ω**|**Ω**] refers to the four-center electron repulsion integrals (ERIs) defined over quaternion overlap distribution functions Ω_μν_ = Ω_μν_(**r**) ≡ **X**_μ_^†^(**r**)**X**_ν_(**r**) in terms of which one can design an efficient algorithm for the evaluation of relativistic ERIs facilitating quaternion algebra and restricted kinetic balance (RKB) conditions^84^ for quaternion basis functions **X**(**r**).^85^ The xc kernel (**K**^xc^) in eq 6 is defined in our closed-shell DR-TDDFT implementation in a noncollinear fashion as described in ref (63). In addition, the adiabatic approximation^86^ of TDDFT is assumed, resulting in a frequency-independent xc kernel. For more details of the relativistic 4c methodology including quaternion RKB basis, integral evaluation, and noncollinear xc potential and kernel as employed in the RESPECT program, we refer to ref (85).

The DR-TDDFT equation (eq 4) is solved using an iterative subspace algorithm that represents the response vectors in the form of the linear combination of trial vectors, since the size of the matrix on the left-hand side of the equation prohibits its direct inversion or the use of elimination techniques for realistic molecular systems. The iterative subspace solver adapted for relativistic 4c-DR-TDDFT requires a proper parametrization of the response equation based on hermicity and time-reversal symmetry.^63,87^ Moreover, a suitable noncollinear kernel and careful control of the numerical precision of the algebraic operations are required to achieve stable convergence.^63^ As indicated by eq 2, the frequency dependence of the spectral function enters via the response vectors. Therefore, the DR-TDDFT equation, eq 4, has to be solved for each frequency within the spectral range of interest to obtain the corresponding response vectors. However, instead of solving each equation separately in a sequential manner, the DR-TDDFT solver in RESPECT works in a multifrequency regime that allows several frequencies (tens to hundreds) to be considered simultaneously in a common trial subspace, thus covering a large part of the spectrum in a single run and reducing the computational demands. The details of the solution of the DR-TDDFT equation for closed-shell systems as implemented in RESPECT can be found in ref (63).

An integral part of the DR-TDDFT calculations of X-ray spectra in atom-centered basis sets is an isolation of core-level excitations by means of a REW-like approach. This is due to intruder transitions arising from electronic excitations from valence (or higher-lying inner shells) to high-lying (above-ionization threshold) virtual orbitals, which are nonphysical in calculations employing finite atom-centered basis sets that cannot accurately describe continuum states. When these transitions fall into the spectral range of interest, they appear as artifacts in XAS simulations. While one way of identifying these transitions is to recalculate the spectra in different basis sets, as the spurious nonphysical peaks are much more sensitive to changes in the basis set, it is not considered practical and a molecular orbital-energy-based cutoff for virtual orbitals has been suggested instead.^72^ Moreover, the REW approach has been applied in nonrelativistic RT-TDDFT^43^ where the same problem with spurious peaks appears. In the present DR-TDDFT work, we also employ a technique first introduced in the context of RT-TDDFT.^18^ It is based on changing the values of some elements of the electric dipole moment operator **P** (on the right-hand side of eq 4). Namely, only those elements of the electric dipole moment operator *P*_*ãĩ*_ that correspond to the targeted core occupied orbitals *ĩ* and sufficiently many virtual orbitals *ã* to cover the desired spectral range are kept at their original value while the rest of the elements of **P** are set to zero. Mathematically, the DR-TDDFT equation, eq 4, represents a large system of linear equations, and this prescription sets the right-hand side of some of these equations to zero while keeping these equations in the system and not removing them. The damped response equation is thus solved in its full dimensions, which preserves orbital relaxation effects. An interpretation of this procedure is that the nonphysical transitions in the spectral range of interest were artificially made dipole forbidden.

## 3 Computational Details

For the purpose of a benchmark and calibration study, a set of closed-shell heavy-metal compounds with available high-quality experimental data were selected. These involve 3d, 4d, 5d, and 5f elements with various electronic configurations of the central atom, namely, VOCl_3_, CrO_2_Cl_2_, MoS_4_^2–^, WCl_6_, PdCl_6_^2–^, ReO_4_^–^, and UO_2_(NO_3_)_2_. In addition, XAS spectra of larger systems, namely, [RuCl_2_(DMSO)_2_(Im)_2_] and [WCl_4_(PMePh_2_)_2_], with DMSO, Im, and Ph standing for dimethyl sulfoxide, imidazole, and phenyl, respectively, were calculated to assess the optimized computational protocol.

All molecular geometries were optimized using a protocol designed for transition metal complexes:^88,89^ PBE0 functional^90−93^ and def2-TZVPP basis sets^94^ for all atoms (def-TZVP in the case of the uranium complex) with the corresponding effective core potentials (ECPs)^95^ replacing 28 core electrons in 4d and 60 electrons in 5d and 5f elements using the TURBOMOLE quantum-chemical program.^96^

All X-ray spectra were calculated using a damped response library^63^ of the Relativistic Spectroscopy DFT program RESPECT(85) and uncontracted all-electron GTO basis sets. For the basis set calibration, we selected the uncontracted Dyall’s VXZ (X = D, T)^97−101^ basis set for metals and the uncontracted Dunning’s cc-pVXZ (X = D, T) and aug-cc-pVXZ (X = D, T)^102−104^ basis sets for light elements. For the calibration of exchange–correlation (xc) functionals, we tested the pure generalized gradient approximation (PBE^90,91,93^), global hybrids (PBE0,^90−93^ B3LYP^93,105−108^), global hybrids (B3LYP-*X*HF, PBE0-*X*HF) with variable exact-exchange admixture *X* (ranging from 0% to 60%), and range-separated hybrid (CAM-B3LYP^93,105−109^) xc functionals. The numerical integration of the noncollinear exchange–correlation potential and kernel was done with an adaptive molecular grid of medium size (program default).

In all 4c calculations, atomic nuclei of finite size were approximated by a Gaussian charge distribution model.^110^ Moreover, the four-center two-electron repulsion integrals were treated within an atom-pair approximation where all integrals over the atom-centered small-component basis functions *X*^S^ were discarded unless the bra and ket basis pairs shared the same origin, i.e., [*X*_A_^S^*X*_B_^S^|*X*_C_^S^*X*_D_^S^]δ_AB_δ_CD_. Excitations to virtual negative-energy states were neglected in the damped response calculations at the 4c relativistic level of theory (no-virtual-pair approximation). A validity test of this approximation is available in Section S1.1.6.

The damped linear response calculations covered the spectral regions with a resolution of 0.1 eV. When plotting the calculated spectra, smoothing by means of B-spline interpolation was used. All linear response calculations employed the multifrequency solver with 50 to 100 frequencies treated simultaneously. The damping/broadening parameter γ used in the damped response calculations was set to 0.15 eV when calibrating and when aiming for high-resolution spectra, while values taken from reference works were used to obtain wider peaks to facilitate the comparison with experimental line shapes, specifically (in eV): 0.5 for VOCl_3_ and CrO_2_Cl_2_, 2.0 for ReO_4_^–^, 0.4 and 1.6 for UO_2_(NO_3_)_2_, and 3.0 for WCl_6_ and [WCl_4_(PMePh_2_)_2_]. For all calculations, the elements of the perturbation operator corresponding to the target occupied (p orbitals for L-edges, d orbitals for M-edges) and all virtual orbitals were preserved, while the remaining elements were discarded (zeroed out). Exceptions were PdCl_6_^2–^ and UO_2_(NO_3_)_2_ where only a subset of the core p and d orbitals was maintained as discussed in detail in Section S1.1.4.

## 4 Results and Discussion

### 4.1 Calibration

In this section, we first investigate the role of the molecular geometry, basis set, and exchange–correlation functional for 4c relativistic DR-TDDFT calculations of XAS spectra. On the basis of this calibration study, we determine a computational protocol that is subsequently validated on larger molecular systems and used to resolve broad signals in their experimental XAS spectra, thus providing their ligand field theory-based interpretation.

#### 4.1.1 Role of Geometry Optimization

We first briefly assess whether to use optimized or experimental geometries. MoS_4_^2–^ has one of the largest differences between the X-ray structure and the geometry optimized at the PBE0/TZVPP level of theory of the whole test set and was therefore selected for comparing calculated XAS spectra at these two geometries. Whereas the experimental X-ray structure has C_3v_ symmetry with Mo–S bonds of 2.193 and 2.172 Å as a result of crystal packing, the PBE0/TZVPP geometry was optimized in vacuo and has T_d_ symmetry with all Mo–S bond lengths being 2.193 Å. The M_4,5_- and L_2,3_-edge spectra were calculated using the PBE0 functional and the uncontracted Dyall’s VDZ basis set for Mo and the uncontracted Dunning’s aug-cc-pVDZ basis for sulfur. The differences between the X-ray and optimized structure in the L- and M-edge spectra were negligible with no significant differences in positions and intensities of the main absorption bands. Some minor peaks of low-intensity in the M-edge spectra were shifted by ∼0.1 eV, which is at the limits of the numerical accuracy of our calculations (figures with the calculated spectra are available in Section S1.1.1). Since the calculated XAS spectra show only a minor dependence on the choice of geometry, including differences in symmetry, PBE0/TZVPP-optimized geometries were used in all subsequent calculations.

#### 4.1.2 Role of Basis Set

The effect of the basis set on XAS spectra was investigated by comparing calculations performed with different uncontracted Dyall’s VXZ (X = D, T) basis sets for the central heavy-metal atoms and with different uncontracted Dunning’s cc-pVXZ and aug-cc-pVXZ (X = D, T) basis sets for the ligand atoms. The following basis combinations were selected: VDZ/VDZ, VDZ/aVDZ, VTZ/VTZ, and VTZ/aVTZ, where the first basis refers to the central heavy atom and the second, to the remaining light elements, while the acronym “a” stands for basis set augmentation.

**Figure 1:**
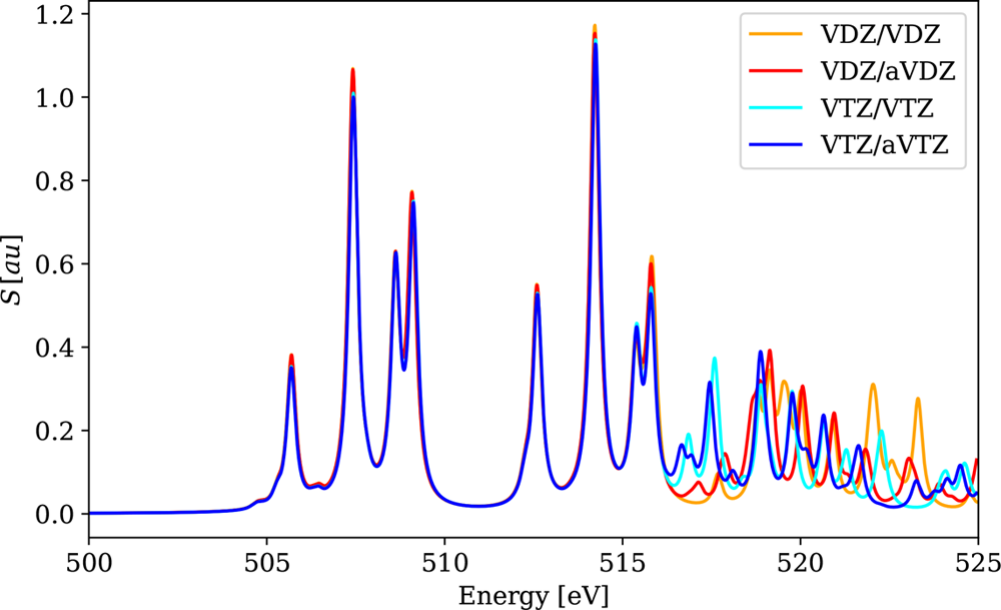
Calculated 4c-DR-TDDFT (PBE0) XAS spectra near vanadium L_2,3_-edges and oxygen K-edges of VOCl_3_ using different basis sets. Basis set notation is defined in Section 4.1.2.

**Figure 2:**
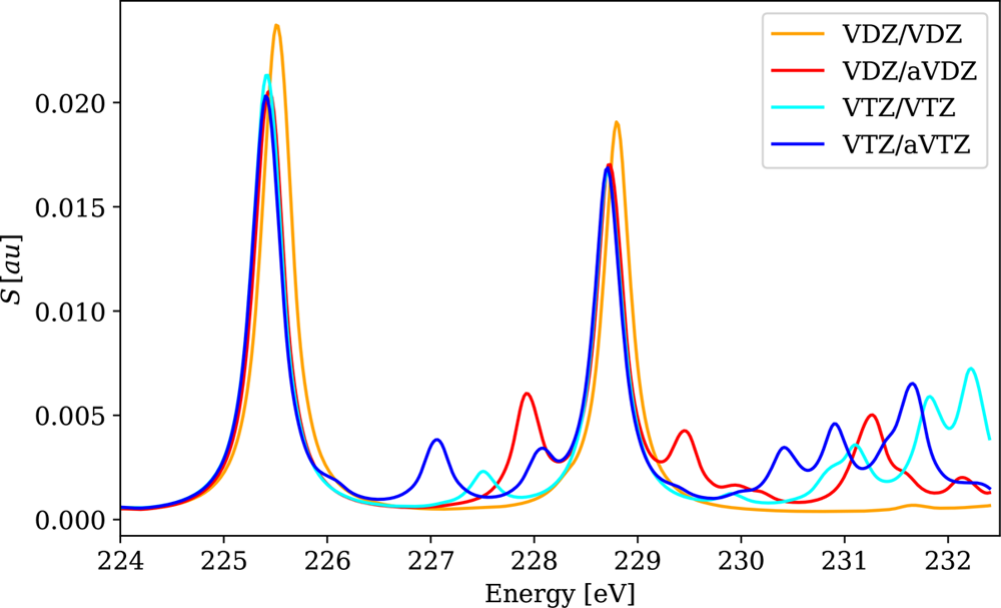
Calculated 4c-DR-TDDFT (PBE0) XAS spectra near molybdenum M_4,5_-edges of MoS_4_^2–^ using different basis sets. Basis set notation is defined in Section 4.1.2.

**Figure 3:**
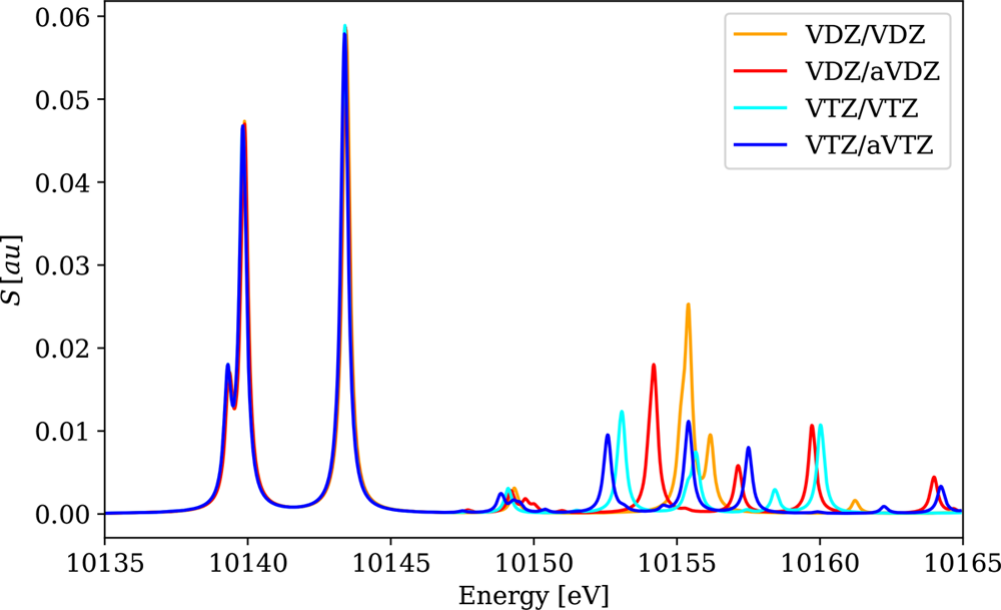
Calculated 4c-DR-TDDFT (PBE0) XAS spectra near the tungsten L_3_-edge of WCl_6_ using different basis sets. Basis set notation is defined in Section 4.1.2.

The results for the main absorption bands are collected in Table 1, and the selected spectra (VOCl_3_, MoS_4_^2–^, WCl_6_) are shown in Figures 1–3. These results show that neither increasing the basis set size from VDZ to VTZ nor augmentation has any significant (>0.1 eV) effect on the positions of the main absorption peaks. However, the number of minor low-intensity signals increases with the size of the basis set, and notable differences are also observed in high-frequency parts of the plotted spectra. However, in some cases, such as the W L_3_-edge in WCl_6_ (Figure 3), these minor peaks lie above the ionization limit and are therefore *not* part of the NEXAFS spectral region. It is therefore advisable to analyze the spectral regions above the ionization limits with caution, in particular in cases when calculations were performed with conventional atom-centered basis sets. Nevertheless, the NEXAFS region of WCl_6_ can be considered as being equally well described in all tested basis sets. The VDZ/aVDZ combination was therefore selected for further calculations because (i) VDZ-based calculations reproduce the main absorption line(s) of the VTZ ones well, but with significant computational savings, (ii) the use of the aVDZ basis for light atoms provides somewhat better agreement with the reference VTZ-based calculations; see for instance, the comparison of the results for different basis sets in the M_4,5_ absorption edges of MoS_4_^2–^ in Figure 2.

**Table 1:** XAS Main Lines (in eV) from 4c-DR-TDDFT (PBE0) Calculations Using Different Basis Sets^a^

		VDZ/VDZ	VDZ/aVDZ	VTZ/VTZ	VTZ/aVTZ
VOCl_3_	L_3_	507.5	507.4	507.5	507.4
	L_2_	514.2	514.2	514.2	514.3
CrO_2_Cl_2_	L_3_	571.4	571.4	571.5	571.6
	L_2_	579.5	579.5	579.5	579.5
MoS_4_^2–^	L_3_	2489.4	2489.3	2489.3	2489.3
	L_2_	2595.9	2595.9	2596.0	2595.9
	M_5_	225.5	225.4	225.4	225.4
	M_4_	228.8	228.7	228.7	228.7
PdCl_6_^2–^	L_3_	3138.2	3138.2	3138.2	3138.2
	L_2_	3297.4	3297.4	3297.6	3297.6
WCl_6_	L_3_	10139.9	10139.9	10139.8	10139.8
	L_2_	11492.7	11492.7	11492.7	11492.7
ReO_4_^–^	L_3_	10472.0	10472.0	10472.0	10472.0
	L_2_	11912.0	11911.9	11912.0	11912.0
UO_2_(NO_3_)_2_	M_5_	3515.3	3515.3	3515.3	3515.3
	M_4_	3693.2	3693.2	3693.2	3693.2

a Basis set notation is defined in Section 4.1.2.

#### 4.1.3 Role of xc Functional Type

**Figure 4:**
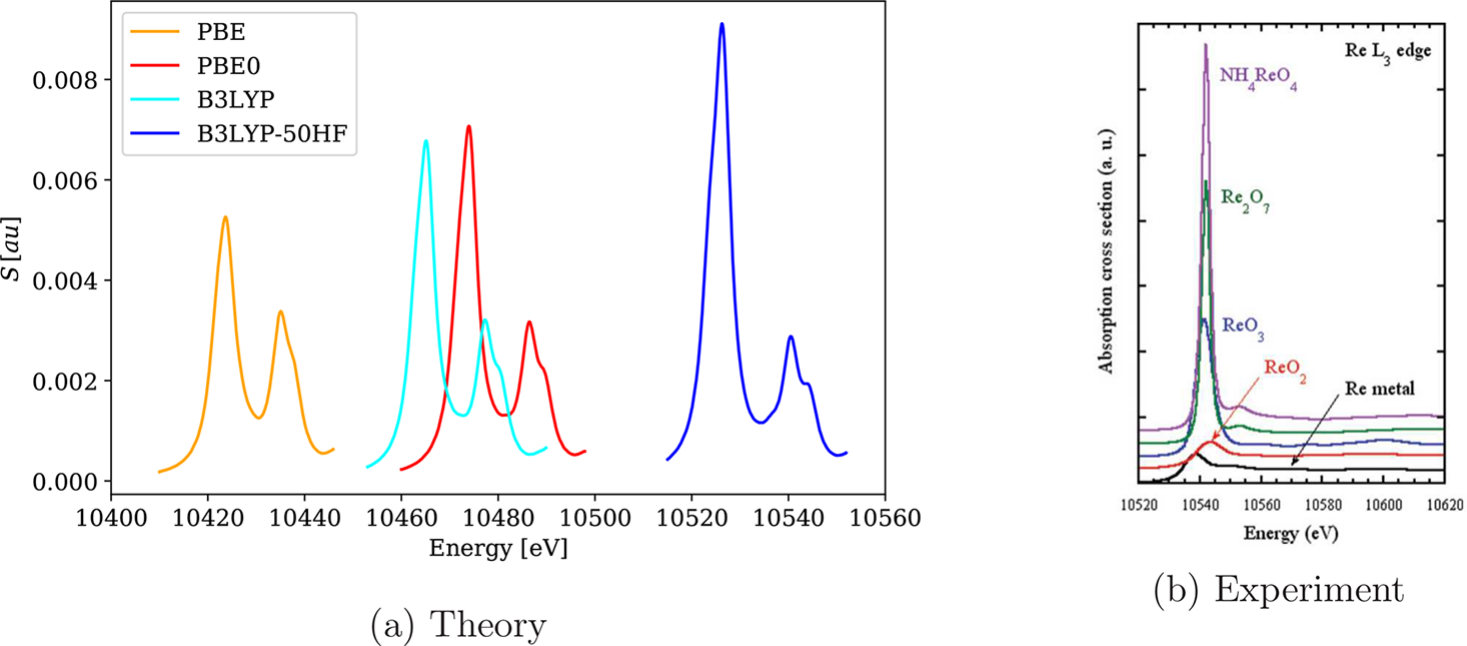
Calculated 4c-DR-TDDFT (VDZ/aVDZ, γ = 2.0) XAS spectra near the rhenium L_3_-edge of ReO_4_^–^ using different xc functionals and their comparison with experimental results.^111^ (a) Theory and (b) experiment (reprinted from ref (111) with permission from APS).

Using the VDZ/aVDZ basis set, we calculated the XAS spectra of the test systems using the PBE, PBE0, B3LYP, B3LYP-50HF, and CAM-B3LYP xc functionals. Unlike for the basis sets, the choice of xc functional significantly affects the position of the absorption edges, as illustrated for the rhenium L_3_ absorption edge of ReO_4_^–^ in Figure 4. The positions of absorption line maxima observed for the tested functionals are reported in Table 2. We see that the calculated results in general underestimate the excitation energies, where PBE provides the worst estimate of the spectral line positions. Significant improvements are achieved by hybrid functionals with the best estimate being given by B3LYP-50HF. Moreover, the range-separated CAM-B3LYP functional leads to almost identical findings as its global hybrid counterpart B3LYP.

**Table 2:** XAS Main Lines (in eV) from 4c-DR-TDDFT (VDZ/aVDZ) Calculations Using Different xc Functionals As Well As Differences between L_2_- and L_3_-Edges (M_4_- and M_5_-Edges) Corresponding to SO Splitting and Their Comparison with Existing Experimental Results

				PBE0		B3LYP		
		exp.^a^	PBE	25HF	40HF	20HF	50HF	CAM-B3LYP
VOCl_3_	L_3_	516.9	499.2	507.5	512.1	506.0	515.6	506.0
	L_2_	523.8	505.9	514.2	518.9	512.8	522.5	512.8
	L_2_–L_3_	6.9	6.7	6.7	6.9	6.8	6.9	6.8
CrO_2_Cl_2_	L_3_	579.9	562.2	571.4	576.6	569.8	580.4	569.8
	L_2_	588.5	570.8	579.5	584.8	577.9	588.7	577.9
	L_2_–L_3_	8.6	8.6	8.1	8.2	8.1	8.3	8.1
MoS_4_^2–^	L_3_	2521.7	2466.9	2489.3	2502.9	2485.4	2513.2	2485.1
	L_2_	2626.0^b^	2572.6	2595.9	2609.6	2591.9	2619.9	2591.6
	L_2_–L_3_	104.3	105.7	106.6	106.7	106.5	106.7	106.5
	M_5_	228.7	220.1	225.4	228.9	224.3	231.2	224.7
	M_4_	231.7	223.4	228.7	232.2	227.6	234.5	228.0
	M_4_–M_5_	3.0	3.3	3.3	3.0	3.3	3.3	3.3
PdCl_6_^2–^	L_3_	3177.8	3112.5	3138.2	3155.0	3133.8	3164.8	3133.3
	L_2_	3334.7	3271.6	3297.4	3312.7	3293.0	3324.4	3292.5
	L_2_–L_3_	156.9	159.1	159.2	157.7	159.2	159.6	159.2
WCl_6_	L_3_	10212.2	10090.8	10139.8	10168.6	10130.7	10191.0	10130.0
	L_2_	11547.0	11442.8	11492.8	11522.4	11483.6	11548.9	11483.0
	L_2_–L_3_	1334.8	1352.0	1353.0	1353.8	1352.8	1354.1	1353.0
ReO_4_^–^	L_3_	10542.0	10422.0	10472.0	10504.0	10463.0	10524.0	10462.0
	L_2_	–	11861.3	11911.9	11942.1	11903.2	11965.3	11902.0
	L_2_–L_3_	–	1439.3	1439.9	1438.1	1431.2	1441.3	1441.0
UO_2_(NO_3_)_2_	M_5_	–	3490.1	3515.3	3530.2	3510.6	3540.6	3509.9
	M_4_	3727.0	3667.8	3693.2	3708.2	3688.5	3718.7	3687.8
	M_4_–M_5_	–	177.7	177.9	178.0	177.9	178.1	177.9

a Experimental references: VOCl_3_, ref (61); CrO_2_Cl_2_, ref (61); MoS_4_^2–^, ref (120); PdCl_6_^2–^, ref (112); WCl_6_, ref (121); ReO_4_^–^, ref (111); UO_2_(NO_3_)_2_, ref (122).

b Value taken from Figure 3 in ref (120), rather than Table 1 ibid. due to a misprint.

Because the choice of xc functional affects the positions of absorption lines, it is of interest to investigate whether the spin–orbit (SO) splitting of core p and d orbitals, represented by the relative positions of L_2_ vs L_3_ and M_4_ vs M_5_ absorption edges, changes with xc functional as well. The calculated SO splittings are reported in Table 2. These results show that the experimental SO splittings are in general well reproduced and all xc functionals perform equally well. These results suggest that the correct description of the SO splitting can be ascribed to the relativistic 4c nature of the Hamiltonian used, whereas incorrect absolute positions of absorption edges are likely due to a self-interaction error, a shortcoming of the GGA and hybrid functionals with smaller amounts of HFX.

**Figure 5:**
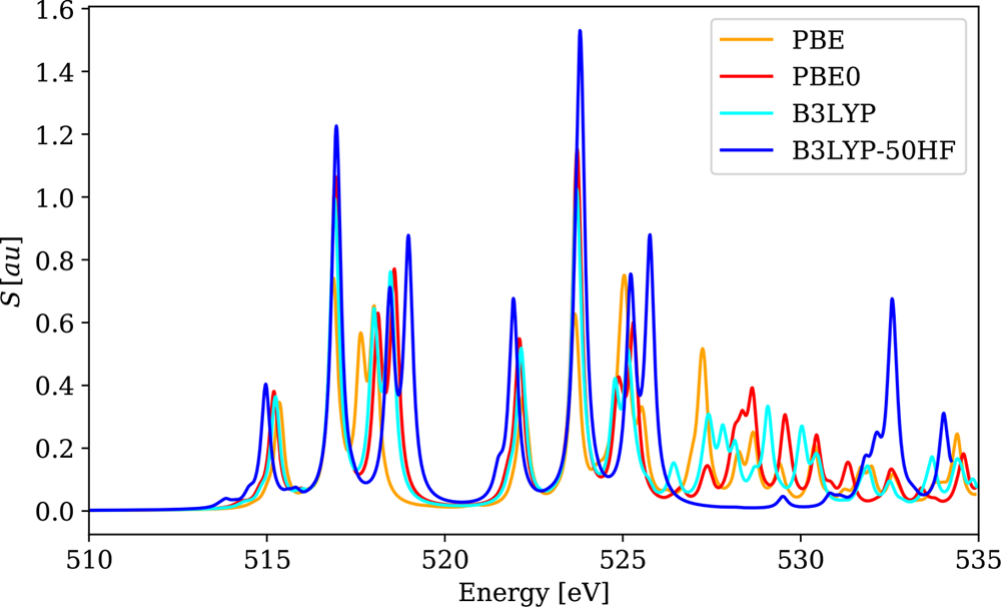
Calculated 4c-DR-TDDFT (VDZ/aVDZ) XAS spectra near vanadium L_2,3_-edges and oxygen K-edges of VOCl_3_ using different xc functionals. To ease comparison, the spectra were shifted so that the L_3_-line maxima are centered on the experimental value.

**Figure 6:**
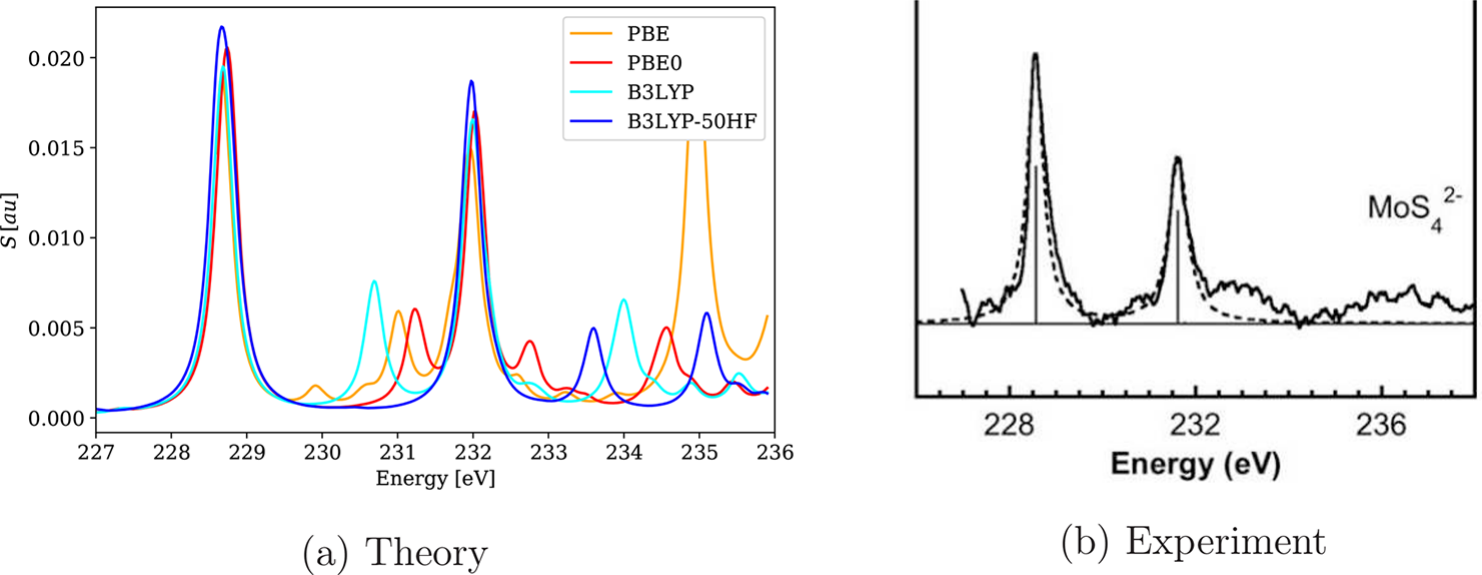
Calculated 4c-DR-TDDFT (VDZ/aVDZ) XAS spectra near molybdenum M_4,5_-edges of MoS_4_^2–^ using different xc functionals and their comparison with experimental results.^120^ To ease comparison, the spectra were shifted so that the M_5_-line maxima are centered on the experimental value. (a) Theory and (b) experiment (reprinted from ref (120) with permission from Elsevier).

The choice of xc functional also affects the overall shape of the spectra, as shown in Figures 4, 5, and 6. In particular, the PBE functional reproduces both energies (see Table 2) and intensities of pre-edge signals poorly with respect to experimental spectra, in addition to overestimating the intensities of the peaks above the edge. This seems to be the case for all GGA functionals; see for instance, the BLYP spectra in Figure S2. Moreover, the range-separated hybrid CAM-B3LYP functional provides spectra similar to global hybrids with smaller admixtures of HFX (B3LYP, PBE0), as shown in Figures S3–S5. The only exception is the M_4,5_ spectrum of MoS_4_^2–^, where the low-frequency minor peaks are not present in the CAM-B3LYP spectrum, thus mirroring the B3LYP-50HF spectrum.

**Figure 7:**
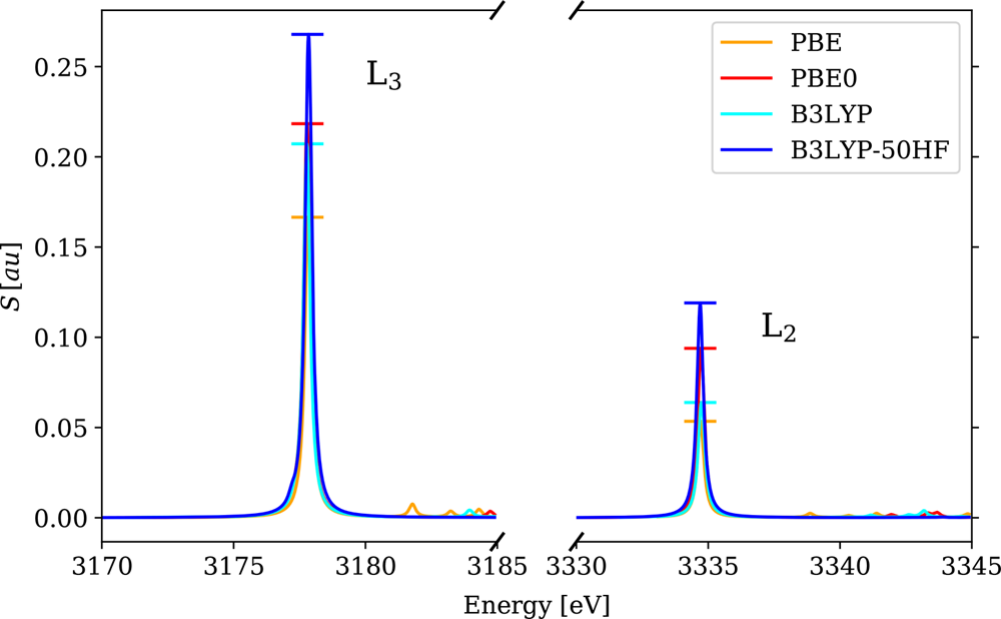
Calculated 4c-DR-TDDFT (VDZ/aVDZ) XAS spectra near palladium L_2,3_-edges of PdCl_6_^2–^ using different xc functionals. To ease comparison, the spectra were shifted so that the maxima of the main lines are centered on the experimental values. The markers denote the intensities of the main lines.

Overall, the functional with the highest amount of HFX (B3LYP-50HF) was found to reproduce experimental spectra the best not only in terms of the lowest absolute energy shift but also in terms of relative signal intensities and positions of minor signals. The correct reproduction L_3_/L_2_ intensity ratio was investigated for the PdCl_6_^2–^ complex, where the value observed in the experiment^112^ is close to the theoretical predicted ratio of 2:1.^7^Figure 7 shows the main peaks near palladium L_3_ and L_2_ absorption edges in PdCl_6_^2–^. The expected intensity ratio of L_3_/L_2_ = 2:1 is correctly reproduced only by B3LYP-50HF, while other functionals yield ratios of 3:1 or even 4:1. Note, however, that branching ratios often differ from expected statistical ratios for a number of reasons.^113,114^ Similarly, the rhenium L_3_ absorption edge in ReO_4_^–^ (Figure 4) has a significantly more intense main line than the only secondary peak for B3LYP-50HF, thus resulting in spectra more consistent with the experiment. Furthermore, spectra calculated with B3LYP-50HF also agree better with the experiments in the fine structure of the spectra; see for example, XAS near the molybdenum M_4,5_-edges of MoS_4_^2–^ in Figure 6, where spectra calculated with other functionals contain minor signals absent in the experimental data. This agrees with earlier observations of the good performance of B3LYP-50HF for core excitations^70^ and led us to perform an additional investigation of the role of HFX in xc functionals.

There exists a good physical ground for the observed trend, which is related to theoretical conditions imposed on xc functionals for several limiting cases. Of relevance for core-level spectroscopies is the high-density limit,^115,116^ which relates to the homogeneous coordinate scaling of the xc functional in the limit of an infinitely large density. In this limit, the exchange energy scales linearly, whereas the correlation energy is known to scale to a finite constant.^117^ Thus, the correlation energy becomes negligible in the high-density limit compared to the exchange energy. In fact, in regions dominated by exchange, that is, where the correlation contribution is considerably smaller than the exchange contribution, the cancellation of self-interaction error (SIE) predominates over the consideration of the nondynamical correlation. Therefore, global hybrid functionals meant to improve the description of core excitations require an alteration in their parametrization, in particular, regarding the amount of admixed exact exchange. Hence, the scaling of HFX can be viewed as correcting for SIE, albeit not entirely satisfying the high-density limit.^118^ A further step to improve this description requires the development and implementation of more sophisticated local hybrid functionals^118,119^ or orbital-specific hybrid functionals such as CVR-B3LYP^74,75^ that, in principle, can satisfy the limiting case. However, the implementation and validation of these specific functionals are beyond the scope of the present work.

#### 4.1.4 Role of the Amount of HF Exchange

**Figure 8:**
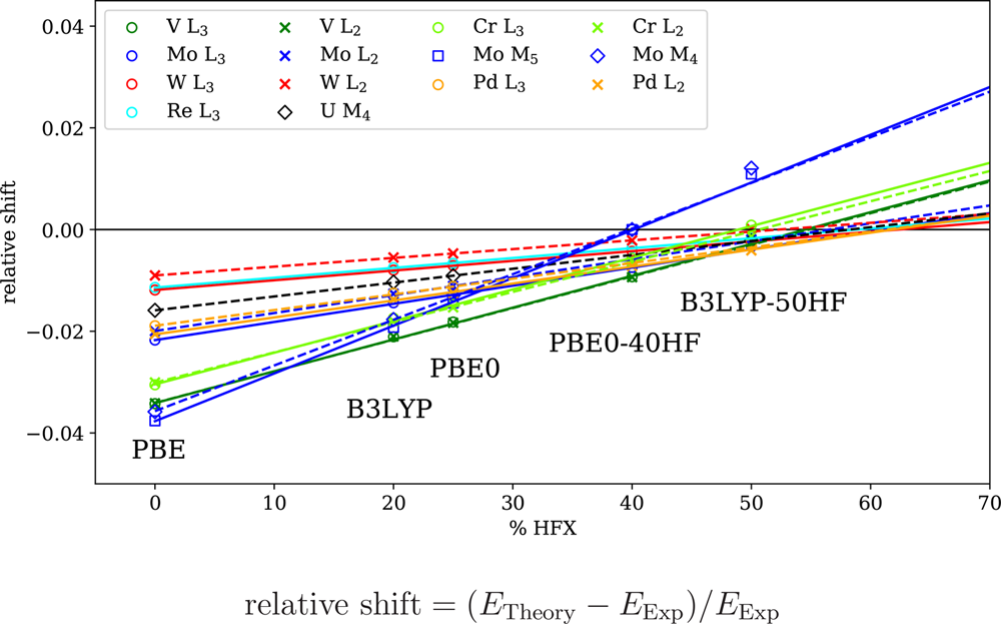
Relative shifts of spectral lines as functions of the percentage of HFX in a functional. For each molecule, a linear fit describes this dependence well and is used to estimate an optimal amount of HFX.

In order to determine the dependence of the XAS spectra on the amount of HFX and to see whether there is an optimal value that best reproduces experimental main lines, we investigated the series of functionals derived from PBE0 and denoted here as PBE0-*X*HF where *X* = 0, 25, and 40. This series was selected due to its good and reliable performance in calculations of the properties of transition metal systems, requiring typically more HFX than the standard PBE0 parametrization.^88,89,123^ We also explored the effect of different amounts of HFX in the CAM-B3LYP functional, going from 65% to 100%. However, this modified range-separated functional gave results almost identical with the original CAM-B3LYP functional and was therefore not pursued any further; see Figures S3–S5. The final results are summarized in Table 2, and the dependence of the relative shift, defined as (*E*_Theory_ – *E*_Exp_)/*E*_Exp_, on the amount of HFX is plotted in Figure 8. The figure also includes B3LYP with its standard parametrization of 20% HFX as well as B3LYP-50HF and shows that the relative shift depends *linearly* on the percentage of HFX, regardless of the type of functional used. Therefore, the most important parameter determining the relative shift remains the amount of HFX, not the type of functional. On the basis of this finding, we determined an optimal amount of HFX (rounded to tens) that best reproduces the experimentally observed position of the main absorption edges. The results obtained with these optimal values of HFX together with the errors with respect to the experiments are collected in Table 3.

**Table 3:** XAS Main Lines (in eV) As Well As Differences between L_2,3_- and M_4,5_-Edges Corresponding to SO Splitting, As Obtained from Experiments and 4c-DR-TDDFT (VDZ/aVDZ) Calculations Using the PBE0-*X*HF Functional, where X Is the Determined Optimal Admixture of HFX

			PBE0-*X*HF		
		exp.^a^	*X*	energy	error
VOCl_3_	L_3_	516.9	50	515.2	–1.7
	L_2_	523.8	50	522.0	–1.8
	L_2_–L_3_	6.9		6.8	–0.1
CrO_2_Cl_2_	L_3_	579.9	50	580.0	0.1
	L_2_	588.5	50	588.2	–0.3
	L_2_–L_3_	8.6		8.2	–0.4
MoS_4_^2–^	L_3_	2521.7	60	2523.1	1.4
	L_2_	2626.0^b^	60	2627.6	1.6
	L_2_–L_3_	104.3		104.5	0.2
	M_5_	228.7	40	228.9	0.2
	M_4_	231.7	40	232.2	0.5
	M_4_–M_5_	3.0		3.3	0.3
PdCl_6_^2–^	L_3_	3177.8	60	3173.4	–4.4
	L_2_	3334.7	60	3332.9	–1.8
	L_2_–L_3_	156.9		159.5	2.6
WCl_6_	L_3_	10212.2	60	10207.3	–4.9
	L_2_	11547.0	60	11561.7	14.7
	L_2_–L_3_	1334.8		1354.4	19.6
ReO_4_^–^	L_3_	10542.0	60	10541.0	–1.0
	L_2_	–	60^c^	11982.1	–
	L_2_–L_3_	–		1441.1	–
UO_2_(NO_3_)_2_	M_5_	–	60^c^	3549.9	–
	M_4_	3727.0	60	3728.0	1.0
	M_4_–M_5_	–		178.1	–

a Experimental references: VOCl_3_, ref (61); CrO_2_Cl_2_, ref (61); MoS_4_^2–^, ref (120); PdCl_6_^2–^, ref (112); WCl_6_, ref (121); ReO_4_^–^, ref (111); UO_2_(NO_3_)_2_, ref (122).

b Value taken from Figure 3 in ref (120), rather than Table 1 ibid. due to a misprint.

c Since an experimental reference was not available, we selected 60% HFX as the most likely optimal value on the basis of other spectra in the calibration.

The optimal amount of HFX is partially dependent on the frequency (energy) of the absorption in question. For absorptions edges above 1000 eV, which include the heavy-element L_2_- and L_3_-edges and the uranium M_4_-edge, the optimal amount of HFX turned out to be 60%. In the case of WCl_6_, the optimal amount was 60% for the L_3_-edge and 50% for the L_2_-edge due to a slightly larger error in the SO splitting and the missing Gaunt term, which was compensated in the fitting procedure by different amounts of HFX. Therefore, we adopt a single value of 60% for the amount of HFX as this provides the best agreement with the experiment across the board, although in some cases, the separation of signals within one spectral range increases with the amount of HFX. While the absolute errors in the W L_2_-edge of WCl_6_ as well as the L_2_ – L_3_ difference with 60% HFX are larger than in all other systems, the relative error remains below 0.2% for absorption line energies and below 2% for the SO splitting, which is a very good result in the context of DFT calculations. This amount of HFX admixture is therefore used in the remaining calculations of heavy-element XAS spectra above 1000 eV.

**Figure 9:**
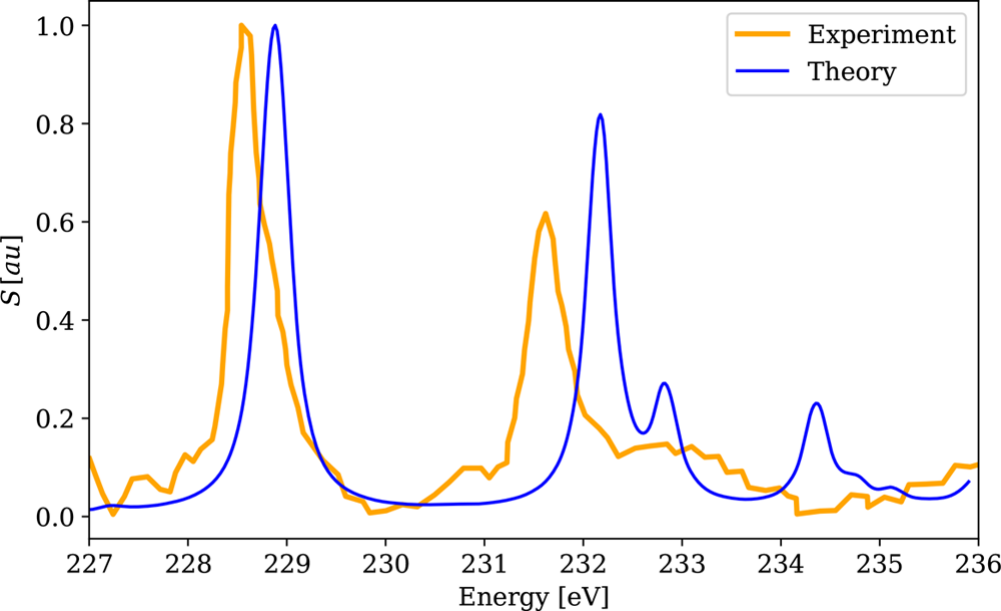
Calculated 4c-DR-TDDFT (PBE0-40HF, VDZ/aVDZ) XAS spectrum near molybdenum M_4,5_-edges of MoS_4_^2–^and its comparison with the experimental results.^120^ The spectra were normalized by setting the maximum to unity. No shift on the energy axis was applied.

Whereas 60% of HFX seems to be quite robust for high-energy spectra, caution should be taken for spectral ranges below 1000 eV, where the optimal amount varies from 50% for V L_2,3_-edges in VOCl_3_ and CrO_2_Cl to 40% for Mo M_4,5_-edges in MoS_4_^2–^. The optimal amount of HFX seems to scale with energy as indicated by 40% needed for ∼200 eV edges and 50% for 500–550 eV edges, suggesting that a simple, e.g., linear, prescription for lower X-ray energies exists, but its exact determination requires further studies. By using a fully relativistic theory and a single optimal amount of HFX for selected energy ranges, we avoid the need for empirical shifts of each spectrum to match the corresponding experimental reference. Such a simple computational prescription offers a very satisfactory agreement between calculated and experimental results. This is demonstrated in Figure 9, and similarly, no shifts are applied to calculated spectra presented onward when comparing them to the experiments.

#### 4.1.5 Role of DR-TDDFT Damping Parameter

**Figure 10:**
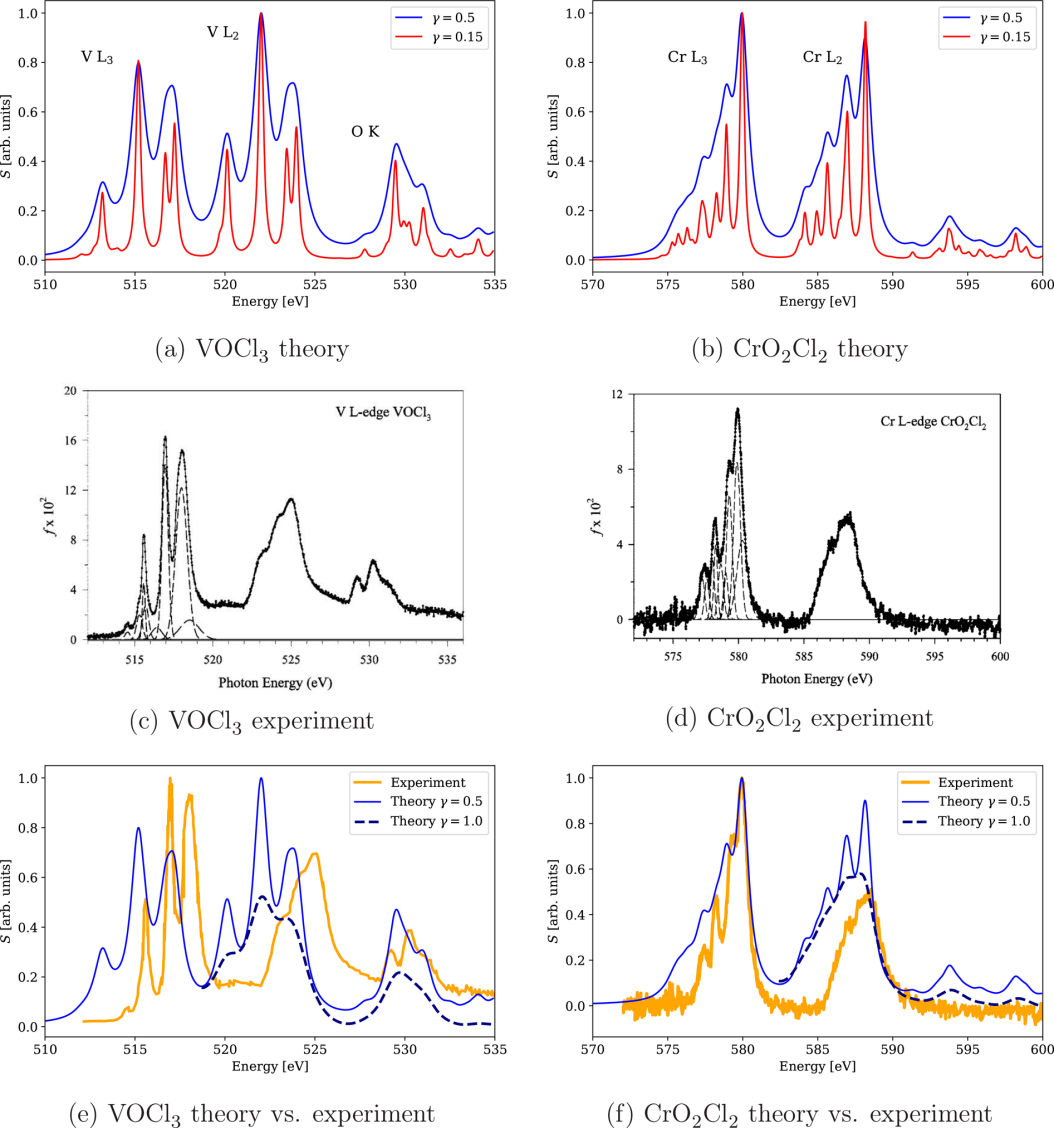
Calculated 4c-DR-TDDFT (PBE0-50HF, VDZ/aVDZ) XAS spectra near vanadium and chromium L_2,3_-edges and oxygen K-edge of VOCl_3_ and CrO_2_Cl_2_ using different damping parameters and their comparison with the experimental results.^61^ The spectra were normalized by setting the maximum to unity with the exception of γ = 1.0 spectra in (e) and (f), which were normalized to preserve the intensity ratio of the maxima for γ = 1.0 and γ = 0.5 as well as shifted on the vertical axis to connect with γ = 0.5 spectra. (a) VOCl_3_ theory, (b) CrO_2_Cl_2_ theory, (c) VOCl_3_ experiment, (d) CrO_2_Cl_2_experiment, (e) VOCl_3_ theory vs experiment, and (f) CrO_2_Cl_2_ theory vs experiment, (c, d) Reprinted from ref (61). Copyright 2009 American Chemical Society.

**Figure 11:**
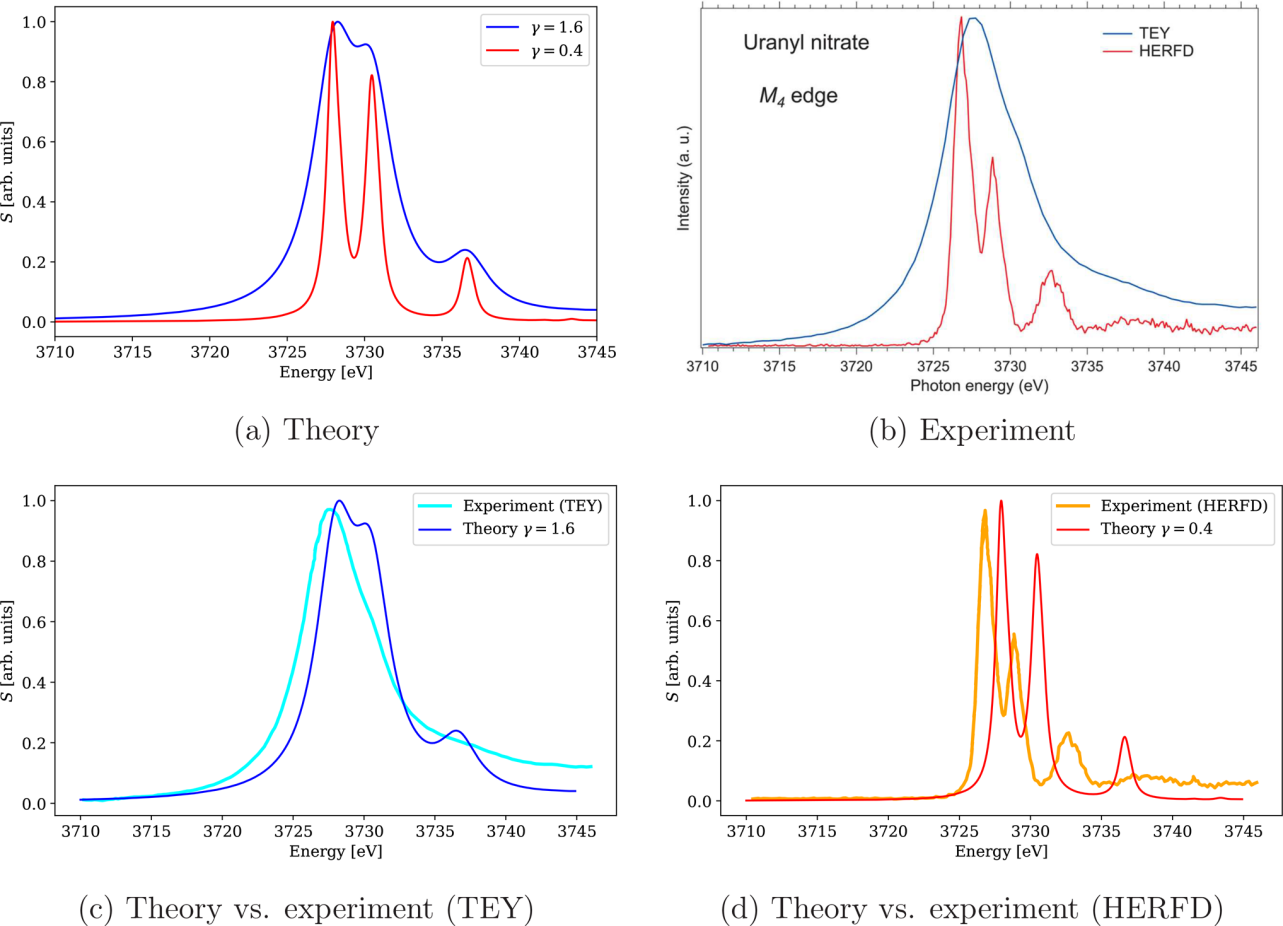
Calculated 4c-DR-TDDFT (PBE0-60HF, VDZ/aVDZ) XAS spectra near the uranium M_4_-edge of UO_2_(NO_3_)_2_ using different damping parameters and their comparison with the experimental results.^122^ The spectra were normalized by setting the maximum to unity. (a) Theory and (b) experiment (reprinted from ref (122), copyright 2016 American Chemical Society). (c) Theory vs experiment (TEY); (d) theory vs experiment (HERFD).

One of the characteristic features of DR-TDDFT is its ability to directly calculate the spectral function for the frequencies of interest without the need to determine the excitation energies–eigenvalues of the Casida equation and their corresponding eigenvectors. The line shape of the spectrum is governed by the damping parameter γ in eq 4 that corresponds to the half width at half-maximum of a Lorentzian peak and models the finite lifetimes of the excited states. Since DR-TDDFT does not provide a means for determining this parameter, a single user-defined damping parameter is used in practice, thus assuming the same lifetime for all excited states that relax back to the electronic ground state. The use of values from available experimental compendia of core-hole lifetimes thus offers a simple way of reproducing experimental lineshapes. Moreover, a decrease in the damping parameter serves as a theoretical tool to obtain narrow peaks with better resolution and thus eases the interpretation of the experimental spectra. An example is the determination of crystal field splitting where the calculation can complement the determination based on second derivatives of experimental spectra. Examples of spectra calculated with different values of γ are shown for VOCl_3_ and CrO_2_Cl_2_ in Figure 10 and for UO_2_(NO_3_)_2_ in Figure 11. These spectra were calculated using the optimal HFX admixture determined in the previous section, i.e., 50% for VOCl_3_ and CrO_2_Cl_2_ and 60% for UO_2_(NO_3_)_2_. Since the damping parameter affects the peak magnitudes, spectra calculated with different values of the damping parameter were normalized by setting their maximum to unity to ease their side-by-side comparison. Reference experimental spectra are well reproduced by the calculations, particularly in terms of absorption energies and overall lineshapes. The comparison of calculated VOCl_3_ and CrO_2_Cl_2_ spectra with the experiment in Figure 10 also demonstrates that different damping parameters may be applied for the L_2_- and L_3_-edges to achieve even better agreement. Particularly, in heavier complexes, individual edges are calculated separately due to the large spin–orbit splitting, which eases the application of different damping parameters compared to cases when these edges overlap.

### 4.2 Application to Larger Systems

**Figure 12:**
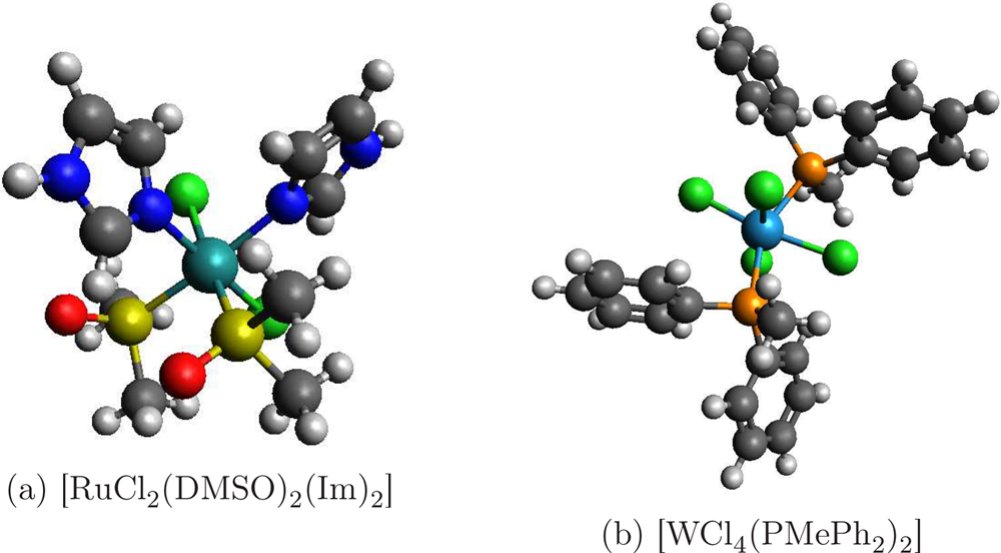
Structures of the larger metal complexes: (a) [RuCl_2_(DMSO)_2_(Im)_2_]; (b) [WCl_4_(PMePh_2_)_2_].

All-electron relativistic 4c DFT calculations of XAS spectra have in the past been limited to small molecules due to their computational cost. However, recent theoretical advances implemented in RESPECT(85) or more specifically in its DR-TDDFT module^63^ have made it possible to tackle XAS of larger and chemically relevant systems. As examples, we employed the calibrated computational protocol (PBE0-60HF, VDZ/aVDZ) and investigated: (a) a ruthenium anticancer prodrug [RuCl_2_(DMSO)_2_(Im)_2_] (Figure 12a) related to chemotherapeutics NAMI-A and KP1019^124^ that consists of 41 atoms and 4652 4c basis spinors; (b) a tungsten complex [WCl_4_(PMePh_2_)_2_] (Figure 12b) used to study the influence of the W oxidation state on XAS spectra^121^ that consists of 59 atoms and 7212 4c basis spinors.

#### 4.2.1 [RuCl_2_(DMSO)_2_(Im)_2_]

**Figure 13:**
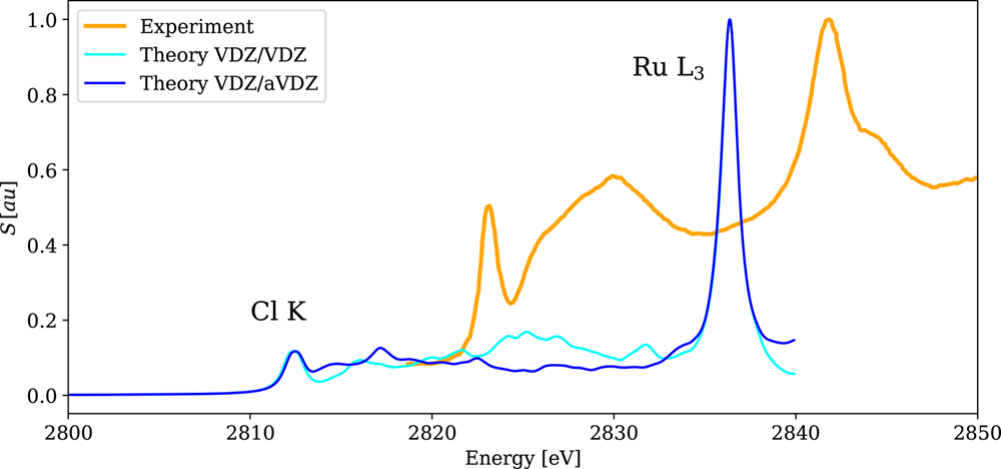
Calculated 4c-DR-TDDFT (PBE0-60HF, γ = 0.5) XAS spectra near chlorine K-edges and ruthenium L_3_-edges of [RuCl_2_(DMSO)_2_(Im)_2_] and their comparison with existing experimental results.^124^

An interesting feature of the XAS spectrum of [RuCl_2_(DMSO)_2_(Im)_2_] is the proximity of the Ru L_3_-edge and the Cl K-edge (Figure 13), which offers a great opportunity to address XAS signals for p- and d-type elements in a single run. The calculated position of the Cl K-edge is 2812.5 eV while that of the Ru L_3_-edge is 2836.4 eV, and they differ from the experiment by −9.8 eV (0.35%) for the Cl K-edge and by −4.7 eV (0.17%) for the Ru L_3_-edge, respectively. In other words, both Cl and Ru XAS transition energies are underestimated, although the difference is rather small considering the total energy of the transitions. A less satisfactory result is obtained when comparing the separation between these edges; the values are 23.9 eV (calculation) vs 18.8 eV (experiment). The energy of the Cl transition is underestimated more than the L_3_-edge of Ru, which is the reason for the increased separation between the edges in comparison with the experiment. This could be due to K-edges at this energy range requiring a different amount of HFX than the Ru L_3_-edge. However, a detailed investigation of this is beyond the scope of this paper.

A very broad peak located between both edges and attributed to transitions from the 1s_1/2_ orbital of Cl to upper-lying virtual orbitals was found closer to the Cl edge in the experiment. While this spectral feature was predicted correctly by calculations with the calibrated computational protocol (PBE0-60HF, VDZ/aVDZ), results obtained with a smaller VDZ/VDZ basis assign its position closer to the Ru L_3_-edge, as seen in Figure 13. At the same time, both basis sets perform equally well for the main XAS edges (their energies and intensities are essentially the same). While the application of the VDZ/VDZ basis was motivated by its relatively good performance for smaller test systems and by an attempt to explore a computationally less expensive scheme for larger systems; this example shows that the basis set augmentation for light elements is manadatory, regardless of the size and complexity of the investigated molecules.

#### 4.2.2 [WCl_4_(PMePh_2_)_2_]

**Figure 14:**
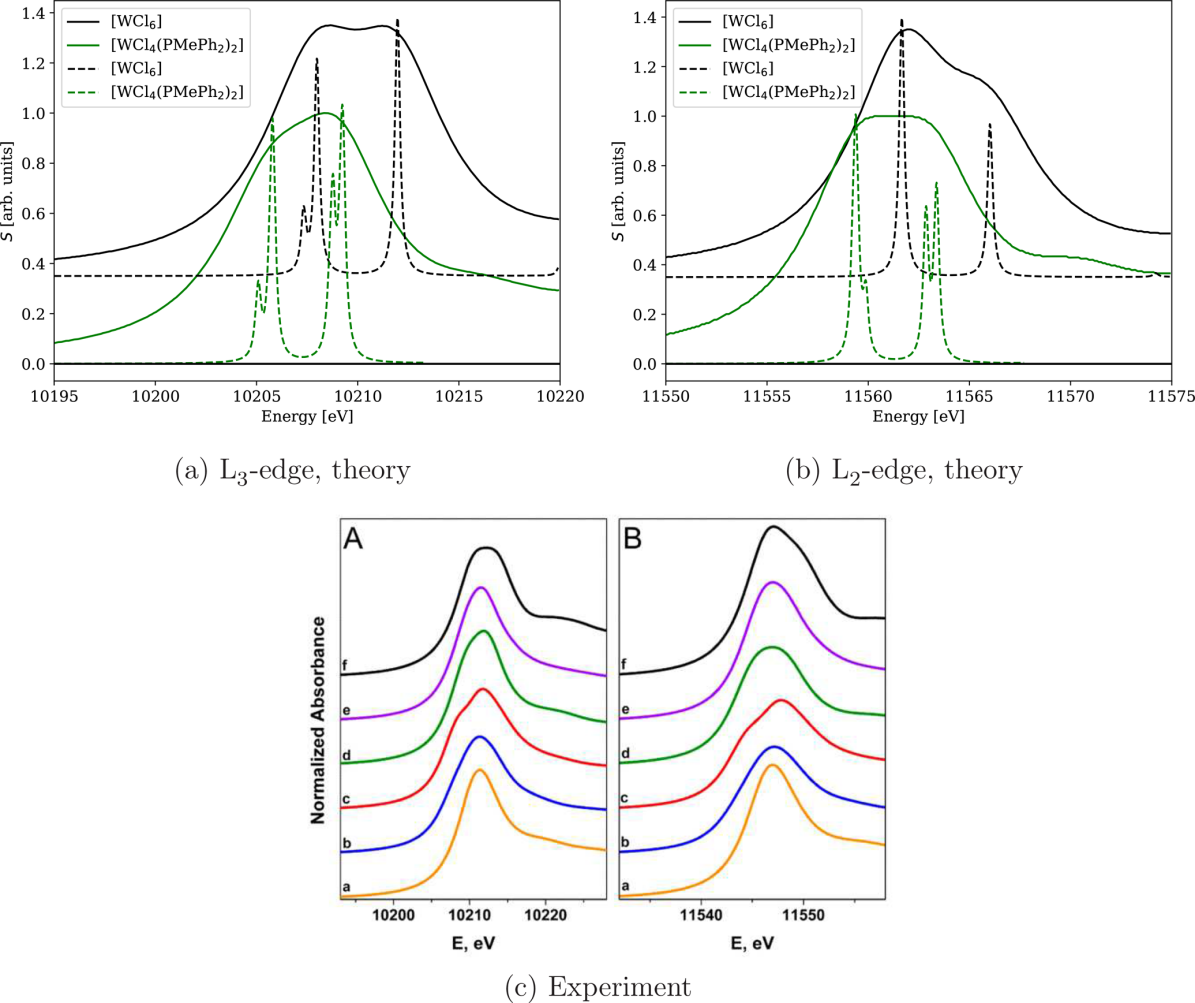
Calculated 4c-DR-TDDFT (PBE0-60HF, VDZ/aVDZ) XAS spectra of WCl_6_ and [WCl_4_(PMePh_2_)_2_] near the tungsten L_3_-edge (a) and L_2_-edge (b) with two different damping parameters, γ = 3.0 (solid line) and γ = 0.15 (dashed line). The compounds correspond to lines f and d in the experimental spectra^121^ (c) (panel A L_3_-edge, panel B L_2_-edge) and are color coded the same way: (a) L_3_-edge, theory, (b) L_2_-edge, theory, and (c) experiment (reprinted from ref (121), copyright 2014 American Chemical Society).

**Figure 15:**
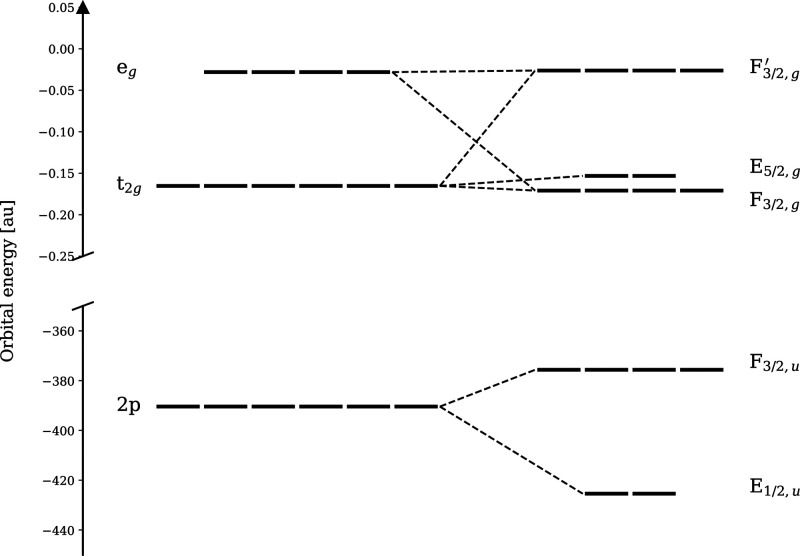
Schematic MO diagram of WCl_6_ showing the assignment of its core and valence spin–orbitals to double point group irreducible representations. The orbital energies without SO coupling were obtained using the scalar-relativistic Douglas–Kroll–Hess Hamiltonian of the second-order. The contributions of the atomic 5d orbitals to the molecular orbitals show the combined effect of the SO interaction and the octahedral ligand field: F_3/2,g_^′^ = d_3/2,±3/2_, d_3/2,±1/2_, E_5/2,g_ = (1/6)^1/2^ d_5/2,5/2_ – (5/6)^1/2^ d_5/2,–3/2_, (1/6)^1/2^ d_5/2,–5/2_ – (5/6)^1/2^ d_5/2,3/2_, F_3/2,g_ = (5/6)^1/2^ d_5/2,5/2_ + (1/6)^1/2^ d_5/2,–3/2_, (5/6)^1/2^ d_5/2,–5/2_ + (1/6)^1/2^ d_5/2,3/2_, d_5/2,±1/2_.

The second complex studied, [WCl_4_(PMePh_2_)_2_], was originally investigated experimentally by Jayarathne et al.^121^ in a study of L_1_-, L_2_-, and L_3_-edge spectra of tungsten complexes with different oxidation numbers. Inspired by this work, we have calculated the L_2_- and L_3_-edge spectra of the closed-shell [W^IV^Cl_4_(PMePh_2_)_2_] complex and compared them with the W^VI^Cl_6_ complex from the test set. Figure 14 shows L_2_- and L_3_-edge spectra of WCl_6_ and [WCl_4_(PMePh_2_)_2_] calculated with two different damping parameters and the experimental spectra of various tungsten species from ref (121). All calculated spectra are normalized such that the most intense peak has unit intensity. A larger damping factor (γ = 3.0) was employed for better resemblance with the experiment; see the black and green lines in Figure 14c for the experimental spectra of WCl_6_ (line f) and [WCl_4_(PMePh_2_)_2_] (line d), respectively. On the other hand, spectra calculated using a smaller damping parameter (γ = 0.15) shown by dashed lines were used to identify individual transitions, which comprise the white lines. As both compounds have roughly an octahedral ligand sphere, these narrow peaks correspond to the transitions from the tungsten 2p shell into its unoccupied d orbital sets. In (nonrelativistic) point-group notation, these orbitals are labeled t_1u_ for occupied and t_2g_ and e_g_ for unoccupied states. In the relativistic case, the correct language for symmetries is provided by double point groups.^125^ Here, the core p_1/2_ and p_3/2_ orbitals belong to representations E_1/2,u_ and F_3/2,u_, respectively. Due to their atomic nature, this is true for both WCl_6_ and [WCl_4_(PMePh_2_)_2_]. The unoccupied orbitals in WCl_6_ belong to F_3/2,g_ (a combination of t_2g_ and e_g_) and E_5/2,g_ (t_2g_) representations; see Figure 15. On the other hand, in [WCl_4_(PMePh_2_)_2_], the octahedral symmetry is broken, which leads to further splitting of F_3/2,g_ while no transitions are symmetry forbidden. In the L_2_-edge spectra of octahedral WCl_6_ (Figure 14b), only two signals at 11561.7 eV (E_1/2,u_ → F_3/2,g_) and 11566.0 eV (E_1/2,u_ → F_3/2,g_^′^) are present. This is due to the fact that only transitions E_1/2,u_ → F_3/2,g_ are allowed while the transition E_1/2,u_ → E_5/2,g_ is forbidden. This is also reflected in the absence of the forbidden transition in the MO analysis (see Figures S11 and S12). The intensity ratio of the observed transitions is 3.26:2 (t_2g_/e_g_), which is close to the ideal 3:2 ratio, but due to the forbidden transition, this does not reflect the number of vacant orbitals. The calculated energy difference of these signals, corresponding to ligand field splitting (LFS), is 4.3 eV, which is higher than the experimental value of 3.4 eV. The larger computed splitting may be due to the increased HFX in the functional (60%), as the LFS calculated using the standard PBE0 functional is 3.6 eV, i.e., much closer to the experimental value. Further investigations are needed to determine whether these observed changes in LFS with different amounts of exact exchange are also manifested in other, similar complexes.

In the L_2_-edge spectrum of WCl_6_, the splitting into two levels corresponds to the expectations from nonrelativistic considerations. However, this is due to the forbidden transition. On the other hand, in the L_3_-edge spectrum, the transition F_3/2,u_ → E_5/2,g_ is allowed. Thus, we see an additional peak in the spectrum resulting from the spin–orbit splitting of the t_2g_ level. Note, however, that the t_2g_ is mixed also in F_3/2,g_^′^.

The presence of two different sets of ligands in [WCl_4_(PMePh_2_)_2_] breaks the octahedral symmetry and lifts the degeneracy of the F_3/2,g_ and F_3/2,g_^′^ levels. This is manifested as an ∼0.5 eV splitting of narrow signals in both the L_2_- and L_3_-edge spectra. Instead of the original three levels F_3/2,g_, E_5/2,g_, and F_3/2,g_^′^ in WCl_6_, five levels (each doubly degenerate) appear now with the lowest being fully occupied due to the lower oxidation number of W in [WCl_4_(PMePh_2_)_2_]. Therefore, in both L_2_- and L_3_-edge spectra, we observe four signals in place of the two and three of WCl_6_.

Let us now compare the spectra of both compounds with respect to the differences in the tungsten oxidation state. According to Jayarathne et al.,^121^ the change of the tungsten oxidation state from IV to VI is accompanied by (a) a shift of both L_2_ and L_3_ white lines toward higher energies; (b) an increase of the absolute intensity of white lines; (c) different peak shapes attributed to the increase of the intensity of transitions into the t_2g_ state compared to the e_g_ state due to the increased number of vacancies in the t_2g_ orbitals. All these effects are also seen in the calculated spectra: (a) the W^VI^Cl_6_ white line peaks are shifted toward higher energies with respect to the [W^IV^Cl_4_(PMePh_2_)_2_] complex (Figure 14); (b) their absolute intensities are higher (about 1.5-times for L_2_ and 1.1-times for L_3_, not shown in the figure due to normalization); (c) the shapes of the calculated peaks are in excellent agreement with the experimentally obtained ones; see Figure 14. However, the interpretation of the observed line shapes in the framework of double group theory, i.e., with the inclusion of relativistic effects, is more involved. The change in overall shape of the broad peaks (full lines in Figure 14) with a change in oxidation state in both L_3_- and L_2_-edges is attributed to the relative increase of the intensity of the lower-energy transitions compared to the higher-energy ones (revealed by the dashed lines in Figure 14). Within an absorption edge, the transitions at higher energy are from core orbitals to four F_3/2,g_^′^ spinors in W^VI^Cl_6_ (see Figure 15) and their four broken symmetry counterparts in [W^IV^Cl_4_(PMePh_2_)_2_]. The transitions at lower energy are from core orbitals to four F_3/2,g_ and two E_5/2,g_ spinors in W^VI^Cl_6_ but only to four spinors in [W^IV^Cl_4_(PMePh_2_)_2_] due to the lowest two spinors being occupied. In the L_3_-edge spectra (Figure 14b), the shift of broad peak intensity maxima toward lower energies from [W^IV^Cl_4_(PMePh_2_)_2_] to W^VI^Cl_6_ can thus be attributed to the increased number of vacancies. However, this argument is problematic in the L_2_-edge spectra (Figure 14b) due to the forbidden transition of E_1/2,u_ → E_5/2,g_. However, its broken symmetry-allowed counterpart transition in [W^IV^Cl_4_(PMePh_2_)_2_] is of comparatively low intensity and appears only as a minor shoulder of the main peak in the dashed-line spectrum. Thus, in the L_2_-edge spectra (Figure 14b), the W^VI^Cl_6_ signal has maximum intensity around the position of the F_3/2,g_ transition, whereas the [W^IV^Cl_4_(PMePh_2_)_2_] signal features a broad plateau on the top. To summarize, the presented four-component scheme could be used not only to calculate spectra of even relatively large and chemically relevant systems but also to facilitate an in-depth analysis of individual signals in the framework of double point groups to explain the observed spectral shapes.

## 5 Conclusions

In this work, we applied our recently developed four-component (4c) relativistic linear damped response time-dependent density functional theory (DR-TDDFT) based on the Dirac–Coulomb Hamiltonian including scalar and SO relativistic effects variationally to calculate the XAS spectra of transition metal and actinide compounds. We performed a calibration to determine the best computational protocol in terms of molecular geometry, basis set, exchange–correlation (xc) functional, and other computational parameters to reproduce experimental spectra of systems with different central atoms, ligands, and oxidation states. The differences between spectra calculated at a computationally optimized geometry and at an experimental geometry were found to be negligible, supporting the use of the optimized geometries in all calculations. While the main absorption lines were already described well using a comparatively small basis set of VDZ quality, a reliable description of the secondary peaks required basis sets of at least augmented VDZ (aVDZ) quality for the light elements.

Hybrid functionals were found to perform better than pure functionals. Range-separated functionals provided no further improvement. The variational inclusion of relativity at the 4c-level of theory allowed the effects of both scalar and spin–orbit relativistic effects on the position and shape of the X-ray spectra to be treated. The SO splittings were reproduced well for all systems. By adjusting the amount of Hartree–Fock exchange (HFX) in the hybrid xc functionals, we found that the shift in absorption energies compared to the experiment depends linearly on the amount of HFX. This linear dependence allowed us to determine an optimal amount of HFX that minimizes the energy shift from the experiment. For most of the absorption bands above 1000 eV, the optimal value was close to 60%, which we propose as a rule of thumb for calculating XAS spectra using relativistic TDDFT. Such a setup yields relative errors below 0.2% and below 2% for line energies and SO splittings, respectively. However, at lower energies, the optimal amount varied from 55% to 40% and caution is advised in these cases. While the increase of the HFX amount has been suggested by previous studies, the present work is the first one performed at the 4c relativistic level of theory and focused on L- and M-edges.

Finally, using the optimized methodology, we calculated XAS spectra of larger systems of chemical interest. For [RuCl_2_(DMSO)_2_(Im)_2_], we have reproduced the spectra in a region where Ru L_3_-edge and Cl K-edge overlap. Furthermore, for L_2,3_-edge spectra of [WCl_4_(PMePh_2_)_2_], we resolved broad experimental peaks into well-separated lines and thus provided a ligand field theory-based interpretation and explained the differences in the spectra of this W^IV^ system and the W^VI^ molecule WCl_6_.

Our results demonstrate the necessity of using multicomponent relativistic methodologies to calculate XAS spectra near metal L- and M-edges dominated by SO coupling. DR-TDDFT allows for the direct calculation of XAS spectra in the frequency domain for a user-defined spectral range. Nonphysical peaks resulting from valence to continuum excitations can be removed in DR-TDDFT by selecting an MO window for which the elements of the perturbation operator remain untouched while the elements outside the window are set to zero. Standard hybrid functionals with scaled HFX and relatively small basis sets of VDZ or aVDZ quality are sufficient to reproduce experimental spectra, making such relativistic calculations feasible even for systems with 50–100 atoms. These results show how the accuracy of DFT-based calculations of XAS spectra can be advanced such that the calculated spectra fall into the same range as experimental ones without the need for large shifts that were required before.

## Supporting Information Available

Further calculated spectra, discussion of MO window for the perturbation operator, and molecular geometries (PDF)
