# Supplementary material for: Accurate X-ray Absorption Spectra near L- and M-Edges from Relativistic Four-Component Damped Response Time-Dependent Density Functional Theory
Source: Inorg Chem. 2021 Dec 27;61(2):830–46. doi: 10.1021/acs.inorgchem.1c02412 (PMC8767545; doi:10.1021/acs.inorgchem.1c02412)
Supplement: Supplementary file 1 — ic1c02412_si_001.pdf [file ic1c02412_si_001.pdf]

Accurate X-ray absorption spectra near L- and M-edges from relativistic four-component damped response TDDFT
Supporting information

Lukas Konecny,[*,†] Jan Vicha,[‡] Stanislav Komorovsky,[¶] Kenneth Ruud,[†] and Michal Repisky[*,†]

†*Hylleraas Centre for Quantum Molecular Sciences, Department of Chemistry, University of Tromsø – The Arctic University of Norway, Tromsø, Norway*

‡*Centre of Polymer Systems, University Institute, Tomáš Baťa University in Zlín, Zlín, Czech Republic*

¶*Institute of Inorganic Chemistry, Slovak Academy of Sciences, Dúbravská cesta 9, SK-84536 Bratislava, Slovakia*

E-mail: lukas.konecny@uit.no; michal.repisky@uit.no

S1 Additional spectra

S1.1 Calibration

S1.1.1 Role of geometry optimization

Figure S1: Calculated 4c-DR-TDDFT (PBE0, VDZ/aVDZ) XAS spectra near molybdenum $M_{4,5}$- and $L_{2,3}$-edges of $MoS_4{}^{2-}$ in optimized (blue line) and experimentally determined (orange line) geometries. See the discussion in Section 4.1.1 of the main article.

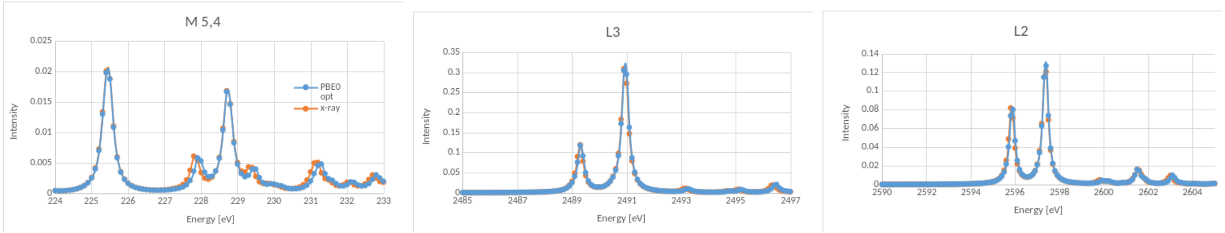

S1.1.2 Pure XC functionals

Figure S2: Calculated 4c-DR-TDDFT (VDZ/aVDZ) XAS spectra using different pure XC functionals.

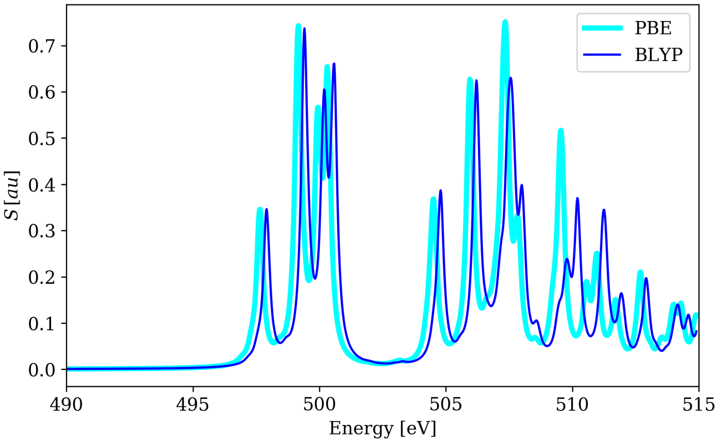

S1.1.3 Range-separated functionals

Figure S3: Calculated 4c-DR-TDDFT (VDZ/aVDZ) XAS spectra near vanadium $L_{2,3}$-edges and oxygen K-edge of $VOCl_3$ using different XC functionals including CAM-B3LYP and CAM100. This is a typical example of CAM-B3LYP performance.

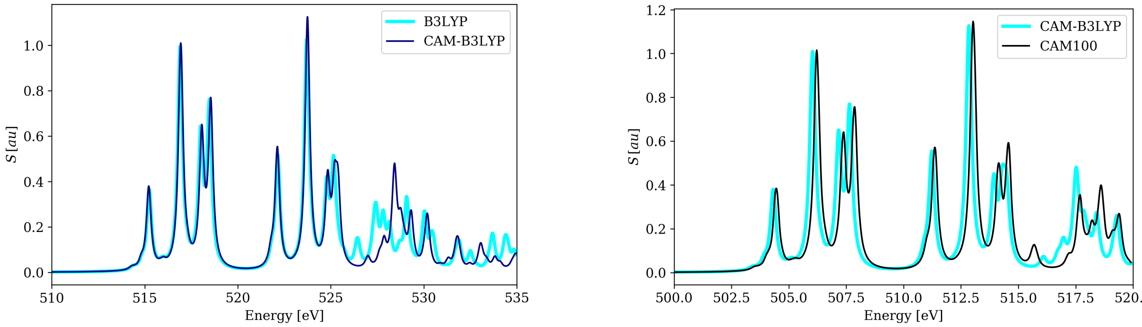

Figure S4: Calculated 4c-DR-TDDFT (VDZ/aVDZ) XAS spectra near molybdenum $M_{4,5}$-edges of MoS_4^{2-} using different XC functionals including CAM-B3LYP and CAM100. This is an example where CAM-B3LYP led to improved line shape similar to B3LYP-50HF while the energy offset was still closer to B3LYP.

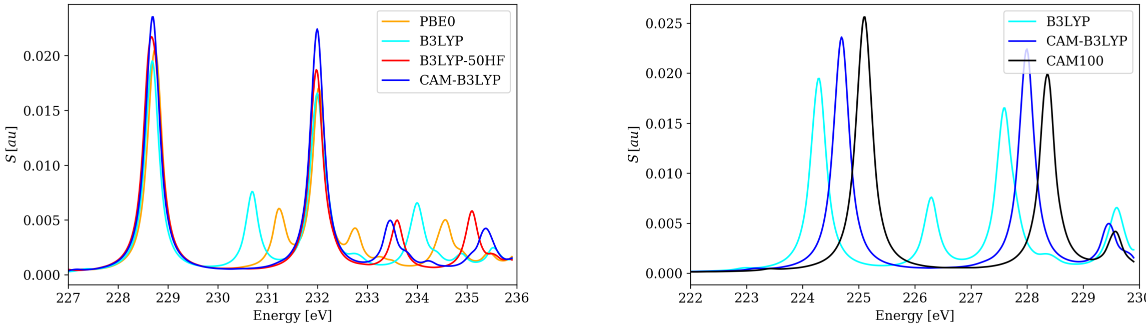

Figure S5: Calculated 4c-DR-TDDFT (VDZ/aVDZ) XAS spectra near rhenium L_3-edge of ReO_4^- using B3LYP, CAM-B3LYP, and CAM100 XC functionals. The range separated functionals do not offer substantial improvement over B3LYP.

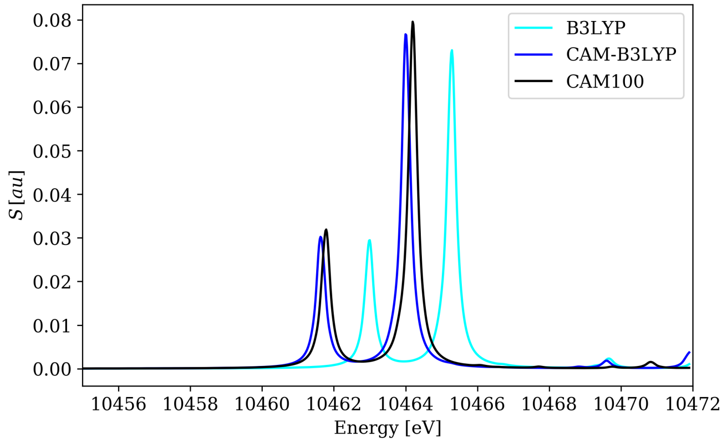

S1.1.4 Role of MO excitation window

All calculations reported in the article use a technique to eliminate spurious valence-to-continuum excitations by zeroing such elements of the matrix representation of the perturbation operator as discussed in the Theory section of the article. A natural choice for the occupied orbitals affected by the perturbation operator is to select all 2p and 3d orbitals for $L_{2,3}$- and $M_{4,5}$-edges, respectively. However, in systems with large spin–obit splitting, the separation between L_2 and L_3 (M_4 and M_5) edges can be so large that transitions from the higher-energy orbitals ($p_{3/2}$ for L_3-edge, $d_{5/2}$ for M_5-edge) occurring within the spectrum originating in excitations from the lower-energy orbitals ($p_{1/2}$ for L_2-edge, $d_{3/2}$ for M_4-edge) lie above the ionization energy, i.e. correspond to excitations to continuum states. As an example see Figure S6 showing L_2-edge spectra of $PdCl_6^{2-}$ using MO window selecting either all p orbitals or only $p_{1/2}$ orbitals. Extra peaks appear in the former case. Because the $p_{1/2}$-$p_{3/2}$ splitting is more than 150 eV for $PdCl_6^{2-}$, for $p_{3/2}$ transitions to appear in the spectral window, $p_{3/2}$ must be excited to above-ionization states that cannot be described in atom-centred basis sets, thus these additional peaks can be proclaimed non-physical. Therefore, in such cases, particularly when using large basis sets, it is recommended to consider only

excitations from a subset of p or d orbitals in order to avoid potential contamination. An alternative approach would be to restrict the virtual orbital space to allow only unoccupied MOs with energies below the ionization energy.

Figure S6: Calculated 4c-DR-TDDFT (PBE0, VTZ/aVTZ) XAS spectra a) near palladium L_2-edge of $PdCl_6{}^{2-}$ using MO window selecting all p orbitals and only $p_{1/2}$ orbitals of Pd; b) near uranium M_4-edge of $UO_2(NO_3)_2$ using MO window selecting all d orbitals and only $d_{3/2}$ orbitals of U.

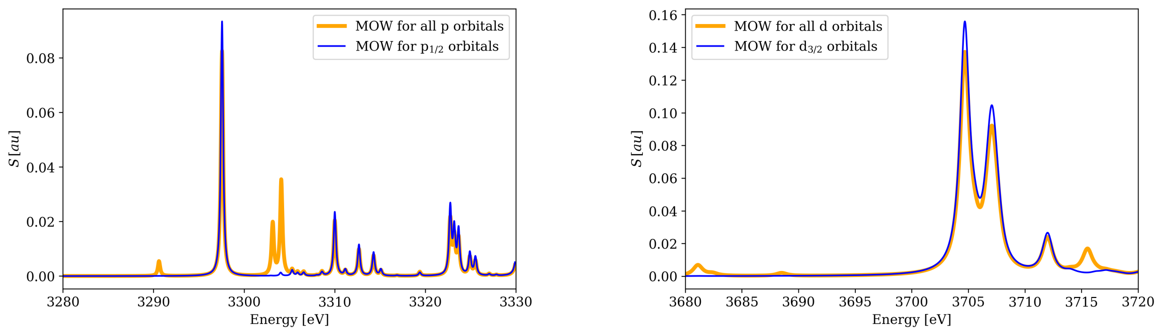

S1.1.5 Spectra without available experimental reference

Figure S7: Calculated 4c-DR-TDDFT (PBE0-60HF, VDZ/aVDZ) XAS spectra near rhenium L_2-edge of ReO_4^-.

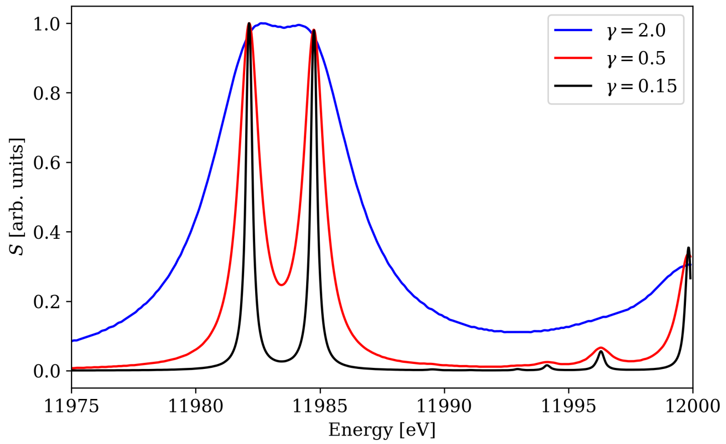

Figure S8: Calculated 4c-DR-TDDFT (PBE0-60HF, VDZ/aVDZ) XAS spectra near uranium M_5-edge of $UO_2(NO_3)_2$.

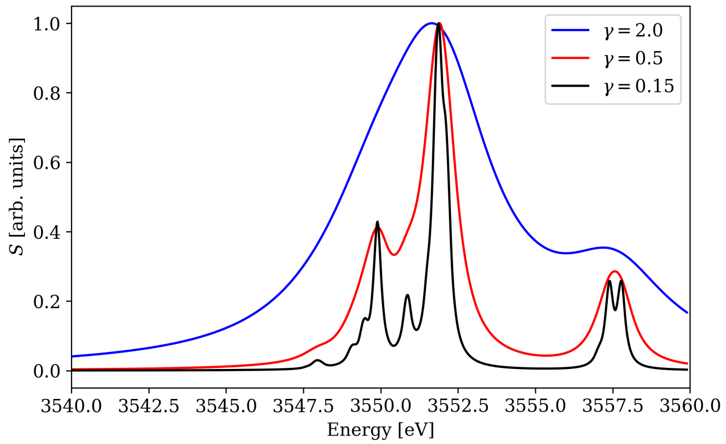

S1.1.6 Effect of transitions between negative- and positive-energy states

Examples of spectra calculated when transition negative- and positive-energy states were and were not considered in 4c-DR-TDDFT.

Figure S9: Calculated 4c-DR-TDDFT (PBE0, VDZ/aVDZ) XAS spectra near rhenium L_3-edge of ReO_4^-.

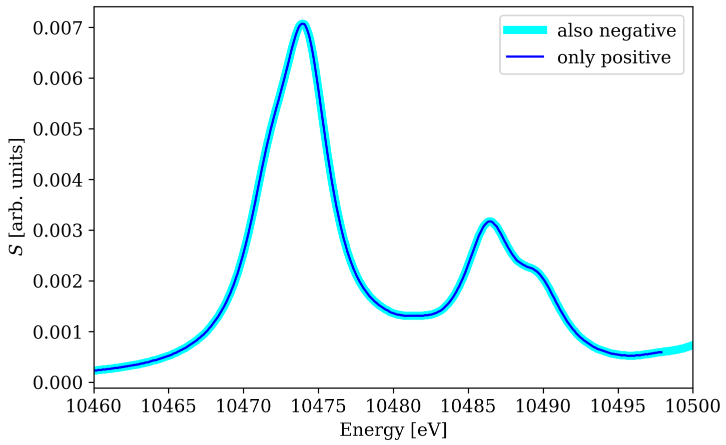

Figure S10: Calculated 4c-DR-TDDFT (PBE0-60HF, VDZ/aVDZ) XAS spectra near tungsten L_3-edge of WCl_6.

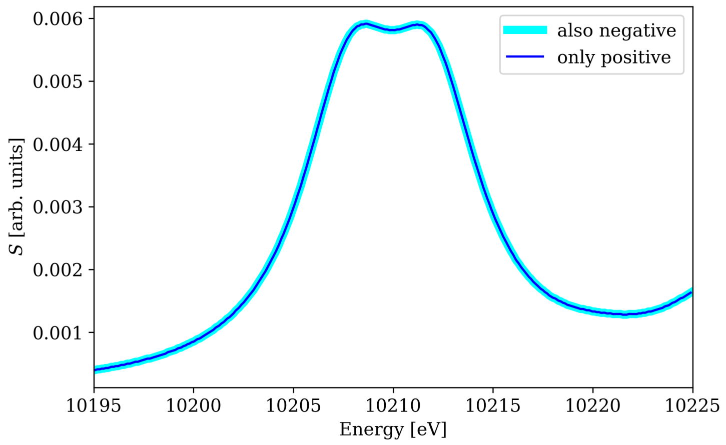

S1.1.7 Double group-based analysis for W systems

The analysis of XAS spectra of W compounds is based on double point groups. Table S1 lists the irreducible representations (irreps) for the relevant spin-orbitals of WCl_6. The irrep of all components of the electric dipole moment is T1u, and its products with the core orbital irrep are

$\mathrm{E}_{1/2,\mathrm{u}} \otimes \mathrm{T}_{1\mathrm{u}} = \mathrm{E}_{1/2,\mathrm{g}} \oplus \mathrm{F}_{3/2,\mathrm{g}}$,

$\mathrm{F}_{3/2,\mathrm{u}} \otimes \mathrm{T}_{1\mathrm{u}} = \mathrm{E}_{1/2,\mathrm{g}} \oplus \mathrm{E}_{5/2,\mathrm{g}} \oplus 2\mathrm{F}_{3/2,\mathrm{g}}$,

showing that the transition $\mathrm{E}_{1/2,\mathrm{u}} \rightarrow \mathrm{E}_{5/2,\mathrm{g}}$ is forbidden.

Table S1: Core and unoccupied orbitals of WCl_6 relevant for XAS near $L_{2,3}$-edges.

orbital	Energy	Double point group irrep.	Point group irrep.
5	-425.4241215	$\mathrm{E}_{1/2,\mathrm{u}}$	$\mathrm{t}_{1\mathrm{u}}$
6	-425.4241215	$\mathrm{E}_{1/2,\mathrm{u}}$	$\mathrm{t}_{1\mathrm{u}}$
7	-375.6558548	$\mathrm{F}_{3/2,\mathrm{u}}$	$\mathrm{t}_{1\mathrm{u}}$
8	-375.6558548	$\mathrm{F}_{3/2,\mathrm{u}}$	$\mathrm{t}_{1\mathrm{u}}$
9	-375.6558547	$\mathrm{F}_{3/2,\mathrm{u}}$	$\mathrm{t}_{1\mathrm{u}}$
10	-375.6558547	$\mathrm{F}_{3/2,\mathrm{u}}$	$\mathrm{t}_{1\mathrm{u}}$
177	-0.1708922	$\mathrm{F}_{3/2,\mathrm{g}}$	e_{g}, $\mathrm{t}_{2\mathrm{g}}$
178	-0.1708922	$\mathrm{F}_{3/2,\mathrm{g}}$	e_{g}, $\mathrm{t}_{2\mathrm{g}}$
179	-0.1708919	$\mathrm{F}_{3/2,\mathrm{g}}$	e_{g}, $\mathrm{t}_{2\mathrm{g}}$
180	-0.1708919	$\mathrm{F}_{3/2,\mathrm{g}}$	e_{g}, $\mathrm{t}_{2\mathrm{g}}$
181	-0.1532279	$\mathrm{E}_{5/2,\mathrm{g}}$	$\mathrm{t}_{2\mathrm{g}}$
182	-0.1532279	$\mathrm{E}_{5/2,\mathrm{g}}$	$\mathrm{t}_{2\mathrm{g}}$
183	-0.0261883	$\mathrm{F}_{3/2,\mathrm{g}}$	e_{g}, $\mathrm{t}_{2\mathrm{g}}$
184	-0.0261883	$\mathrm{F}_{3/2,\mathrm{g}}$	e_{g}, $\mathrm{t}_{2\mathrm{g}}$
185	-0.0261864	$\mathrm{F}_{3/2,\mathrm{g}}$	e_{g}, $\mathrm{t}_{2\mathrm{g}}$
186	-0.0261864	$\mathrm{F}_{3/2,\mathrm{g}}$	e_{g}, $\mathrm{t}_{2\mathrm{g}}$

Figures S11 and S12 show the dominant MO pair contributions to the peaks in L_3- and L_2-edges, respectively (Figures 14 (a) and 14 (b) in the main article). The plots show individual virtual–occupied (ai) MO-pair elements of the dipole polarizability tensor $\alpha_{ai}(\omega) = X_{ai}(\omega)P_{ia} + Y_{ai}(\omega)P_{ai}$, evaluated at the frequency ω corresponding to the energy

of the peak maximum, and where **X** and **Y** are the response vectors and **P** is the matrix representation of the electric dipole moment operator, as discussed in the Theory section of the main article. However, no summation over the indices ai is assumed here. For more details of the analysis, see Konecny, L.; Repisky, M.; Ruud, K.; Komorovsky, S. Relativistic four-component linear damped response TDDFT for electronic absorption and circular dichroism calculations. *J. Chem. Phys.* 2019, **151**, 194112.

Figure S11: MO analysis of spectral transitions in L_3-edge spectrum of WCl_6.

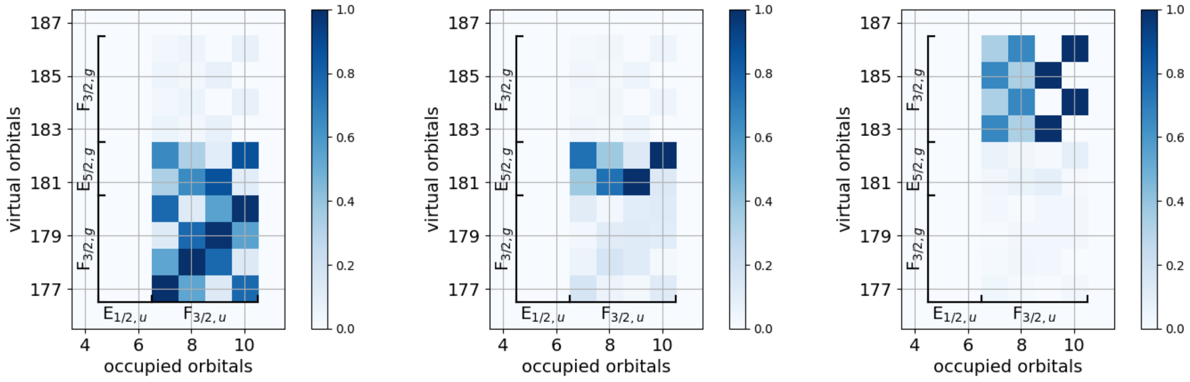

(a) peak1 (b) peak2 (c) peak3

Figure S12: MO analysis of spectral transitions in L_2-edge spectrum of WCl_6.

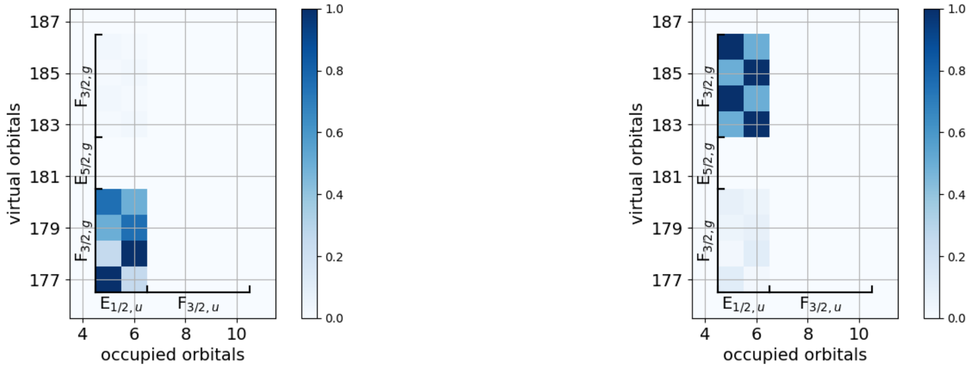

(a) peak1 (b) peak2

S2 Geometries

All molecular geometries are given in Ångström.

Table S2: Molecular geometry of $VOCl_3$.

Atom	x	y	z
V	2.2290187	-1.1797130	-0.0908750
O	2.4041704	-1.0461917	-1.6126195
Cl	0.2242083	-0.6442191	0.3979482
Cl	2.6220625	-3.2042145	0.4493642
Cl	3.6134815	0.1359875	0.8561821

Table S3: Molecular geometry of CrO_2Cl_2.

Atom	x	y	z
Cr	0.88078158	-0.13245116	-0.02959505
Cl	-1.22227445	-0.14808946	-0.05647850
Cl	1.61111615	0.85367740	1.67858777
O	1.39666198	-1.58039095	-0.03621237
O	1.39682473	0.58566316	-1.28685686

Table S4: Molecular geometry of ${MoS_4}^{2-}$.

Atom	x	y	z
Mo	2.4310175	1.7387470	7.0093109
S	2.7198499	1.7387524	4.8370074
S	3.3598198	-0.0499583	7.8694376
S	0.2866474	1.7387525	7.4614107
S	3.3598163	3.5274565	7.8694344

Table S5: Molecular geometry of WCl_6.

Atom	x	y	z
W	3.5315869	2.0389600	5.7670454
Cl	5.1495989	2.9730442	4.4458810
Cl	3.5315673	0.1706769	4.4458067
Cl	3.5315673	3.9072434	7.0882847
Cl	5.1495990	1.1048756	7.0882105
Cl	1.9135701	1.1048759	7.0882103
Cl	1.9135703	2.9730440	4.4458812

Table S6: Molecular geometry of ${PdCl_6}^{2-}$.

Atom	x	y	z
Pd	3.5315810	2.0389600	5.7670462
Cl	5.1848568	2.9932265	4.4169306
Cl	3.5315777	0.1301751	4.4167597
Cl	3.5315774	3.9477441	7.1173315
Cl	5.1848559	1.0846937	7.1171605
Cl	1.8783060	1.0846939	7.1171607
Cl	1.8783051	2.9932267	4.4169307

Table S7: Molecular geometry of ${ReO_4}^{-}$.

Atom	x	y	z
Re	1.4934685	-0.5338437	0.8411442
O	1.6119799	0.5314972	2.1938310
O	0.2220604	-0.0041968	-0.1988355
O	2.9853466	-0.5297356	-0.0263973
O	1.1555746	-2.1330311	1.3952877

Table S8: Molecular geometry of $UO_2(NO_3)_2$.

Atom	x	y	z
U	3.4762959	0.5300554	-0.5098080
N	2.9333958	0.4442629	2.2755600
N	4.0151241	0.6301142	-3.2951080
O	5.1825442	0.4323044	-0.1801379
O	1.7699108	0.6261756	-0.8395763
O	3.1200485	1.5344677	1.6308641
O	3.9361036	1.6737949	-2.5577927
O	2.7066333	0.4085578	3.4373390
O	3.0150766	-0.6036928	1.5447681
O	3.8324350	-0.4638204	-2.6558319
O	4.2358920	0.6727204	-4.4578064

Table S9: Molecular geometry of $[RuCl_2(DMSO)_2(Im)_2]$.

Atom	x	y	z
Ru	1.0823808	1.3041079	0.1880267
Cl	0.7734561	3.6018235	0.8740608
Cl	1.1450420	2.0186901	-2.1182100
C	-1.6567654	0.0998657	-1.4047446
S	-1.1656750	1.0593649	0.0196562
C	-1.9340021	2.5986308	-0.4414959
O	-1.9101692	0.5196407	1.1700987
H	-1.3252009	-0.9200840	-1.2193946
H	-1.1795748	0.5246730	-2.2873559
H	-2.7434568	0.1438438	-1.4709946
H	-1.4323182	2.9790320	-1.3304312
H	-1.7821491	3.2881488	0.3841397
H	-2.9904420	2.3920068	-0.6109234
C	3.0308443	-1.4108433	0.2858381
S	1.4733775	-0.8016002	-0.3646613
C	1.8242665	-1.0940299	-2.0948804
O	0.4878784	-1.8368448	0.0261280
H	2.9634280	-1.3531521	1.3707434
H	3.8599065	-0.8055382	-0.0738677
H	3.1273334	-2.4504412	-0.0258069
H	2.6482235	-0.4590574	-2.4128611
H	0.9382740	-0.8169022	-2.6591589
H	2.0454381	-2.1555655	-2.2038337
N	4.8832852	3.0975981	0.0694091
C	5.1759831	2.3110140	1.1518847
C	4.0826615	1.5223009	1.3294467
N	3.1451298	1.8185636	0.3724159
C	3.6539808	2.7806910	-0.3664411
H	6.1061345	2.3803382	1.6874496
H	3.9070607	0.7843888	2.0917226
H	3.1420966	3.2473721	-1.1920172
H	5.4663956	3.8150351	-0.3215818
N	0.4578474	-0.1818650	4.0845028
C	1.0817823	0.9868169	4.4389115
C	1.4131809	1.5947223	3.2697006
N	1.0010208	0.8071012	2.2216234
C	0.4209525	-0.2533658	2.7452230
H	1.2189723	1.2831078	5.4635236
H	1.8673423	2.5536744	3.0935703
H	-0.0380690	-1.0482396	2.1807274
H	0.0506571	-0.8546136	4.7078172

Table S10: Molecular geometry of $[WCl_4(PMePh_2)_2]$.

Atom	x	y	z	Atom	x	y	z
W	2.1396762	4.2841474	6.1647819	C	2.9271611	-0.9579594	5.5654926
Cl	1.7432161	3.7830972	8.4996792	C	3.8414400	1.1251025	4.7999230
Cl	2.4984758	4.6486696	3.8172660	C	3.3650318	-0.1444191	4.5295200
Cl	0.5911128	2.7270541	5.6001838	H	0.9682672	9.5992083	10.8052290
Cl	3.4956600	6.1846445	6.7347833	H	1.5878126	8.9167095	6.6373039
P	0.5458535	6.1954535	6.2737679	H	1.7666026	10.3765651	8.5953638
P	4.3461574	3.3042894	6.4232453	H	-0.0087443	7.3358963	11.0289461
C	0.6646963	7.2969609	7.7117245	H	-0.1903295	5.8707784	9.0729657
C	1.2228012	8.5665383	7.5930552	H	-1.8661987	7.0788948	4.8053713
C	1.3295597	9.3913517	8.7014390	H	-4.2137335	6.3912794	4.9336621
C	0.8843765	8.9547733	9.9387678	H	-4.9050855	4.6042765	6.4981448
C	0.3345871	7.6874687	10.0640832	H	-3.2096541	3.4899772	7.9164816
C	0.2256896	6.8613490	8.9592635	H	-0.8446056	4.1442594	7.7612522
C	-1.1952714	5.6734336	6.2921825	H	0.4551793	6.7211631	3.9518795
C	-2.1504634	6.2931429	5.4922833	H	1.7582850	7.5755335	4.7607722
C	-3.4798428	5.9062597	5.5652632	H	0.0858605	8.1773708	4.9353337
C	-3.8661818	4.9047461	6.4413484	H	6.7250372	1.7951529	5.8465279
C	-2.9167602	4.2816982	7.2383004	H	8.6741577	2.1986178	4.4002772
C	-1.5870293	4.6561451	7.1604488	H	8.7870891	4.2501892	3.0201927
C	0.7108913	7.2899733	4.8441461	H	6.9427447	5.8934081	3.1030360
C	5.7370639	3.6286595	5.3056270	H	4.3480672	3.1402579	8.8189435
C	6.7786429	2.7025010	5.2557681	H	5.4105744	4.4185146	8.2223403
C	7.8718475	2.9250331	4.4375089	H	5.9572413	2.7238084	8.1293803
C	7.9333789	4.0761702	3.6637900	H	3.4332517	1.1369858	8.1708822
C	6.9003466	4.9981780	3.7107671	H	2.6159478	-1.1321816	7.6821575
C	5.8013669	4.7786234	4.5281046	H	2.5472227	-1.9491450	5.3504252
C	5.0951779	3.3869051	8.0671062	H	3.3251641	-0.4965786	3.5064514
C	2.9667588	-0.5012079	6.8751226	H	4.1576888	1.7740156	3.9923629
C	3.8904257	1.5838555	6.1162153	H	4.9887507	5.4898614	4.5555525
C	3.4411405	0.7688828	7.1534064				
